# Inside the frontal face network: multimodal evidence for distinct emotional and motor resonance circuits

**DOI:** 10.1007/s00429-026-03125-5

**Published:** 2026-05-28

**Authors:** Beatrice De Negri, Luciano Simone, Fausto Caruana, Marzio Gerbella

**Affiliations:** 1 Department of Medicine and Surgery (DIMEC), University of Parma, Parma, Italy; 2 Institute of Neuroscience, National Research Council of Italy (CNR), Parma, Italy

**Keywords:** Mirror system, Prefrontal, Cingulate, Insula

## Abstract

The perception of emotional facial expressions engages widespread cortical networks, extending beyond occipito-temporal visual areas to multiple frontal and perisylvian regions. While the connectivity of the occipito-temporal territories involved in visual processing of faces has been extensively mapped, the frontal and perisylvian pathways, comprising motor, emotional, and cognitive-attentional components, remain less understood. Building on intracranial recordings, electrical stimulation, and cortico-cortical evoked potentials, our previous work identified two frontal-perisylvian clusters: a premotor cluster (constituted by Rolandic operculum and a region between the frontal eye fields and the ventral premotor cortex) supporting motor resonance, and a limbic/prefrontal cluster (composed by prefrontal and cingulate territories) supporting emotional resonance. Here, combining diffusion tractography (DTI) and resting-state fMRI (rsfMRI), we provide a detailed anatomical and functional connectivity framework for these regions. DTI revealed seven principal fiber tracts organizing the clusters into three coherent networks, while rsfMRI demonstrated shared and dissociable connectivity patterns among them partially consistent with Default Mode, Ventral Attention, Dorsal Attention, Frontoparietal, and Sensorimotor networks. The first two networks here identified largely correspond to the limbic/prefrontal and premotor clusters described in our previous study, while the third appears to serve as a nexus between the first two and as a source of visual information for them. These findings outline a tripartite architecture—emotional, premotor, and cognitive networks—through which facial emotion recognition may operates via parallel, functionally specialized streams, enabling both motor and emotional resonance and supporting adaptive social behavior.

**Supplementary Information:**

The online version contains supplementary material available at 10.1007/s00429-026-03125-5.

## Introduction

The passive observation of emotional facial expressions triggers a cascade of neural activations throughout the cortex, not limited to the high-order visual regions of the occipito-temporal face network, but also reaching multiple frontal and perisylvian regions. The existence of multiple, and potentially independent, frontal patches involved in the processing of others’ facial expressions is expected, if one considers the numerous cognitive, emotional and motor processes triggered by emotional faces, which span from automatic emotion recognition to emotional contagion.

Over the years, the combination of different functional and anatomical techniques has led not only to a highly refined identification of the occipito-temporal subregions contributing to the processing of emotional expressions, but also to a clarification of their intrinsic connectivity (Allison et al. 1994; Atkinson and Adolphs 2011; Duchaine and Yovel 2015; Gauthier et al. 2000; Grill-Spector et al. 2017; Haxby et al. 2000; Kanwisher 2000; Pitcher and Ungerleider 2021). Unlike the occipito-temporal visual system, where theoretical models are indeed anchored to robust connectivity data, current knowledge on the frontal contribution to the elaboration of emotional expressions - although solidly demonstrated - does not reach the same level of detail, and the internal organization of frontal face-responsive regions has remained largely unexplored. While existing literature identifies these areas, an integrated framework explaining their intrinsic structural and functional connectivity is lacking.

Lesion studies have demonstrated that the ventromedial prefrontal cortex (vmPFC) and the orbitofrontal cortex (OFC) are essential for detecting and distinguishing emotional expressions (Hornak et al. 1996, 2003; Wolf et al. 2014; Jenkins et al. 2016; Hiser and Koenigs 2018; Rolls 2023). More recent evidence, moreover, indicates that the processing of others’ facial displays is not limited to these prefrontal territories but also involves the inferior frontal gyrus (IFG) and the adjacent anterior insula (AI); Tsuchida and Fellows 2012). Consistent with this, single-cell recordings in nonhuman primates have shown that the prefrontal region between areas 45 and 12 contains face-selective neurons that respond to both facial identity and emotional expressions (Diehl et al. 2022), while, in human, the AI is activated during the observation of facial expressions of disgust and laughter (Wicker et al. 2003; Lombardi et al. 2023; Del Vecchio et al. 2024). These findings, together with the known connectivity of these regions with several sectors of the visual and auditory temporal cortices, suggest that these prefrontal and insular areas may play a crucial role in social communication networks (Gerbella et al. 2010; Borra et al. 2011; Simone et al. 2025). Beyond these prefrontal and insular territories, numerous studies have shown that observing facial actions, including emotional expressions, activates the ventral premotor cortex and the adjacent frontal opercular cortex (Hennenlotter et al. 2005; Niedenthal et al. 2010; Wood et al. 2016; Korb et al. 2023; Del Vecchio et al. 2024).

Research on the mirror neuron system (Wicker et al. 2003; Goldman and Sripada 2005; Goldman 2006; Niedenthal et al. 2010; Rizzolatti et al. 2014; Wood et al. 2016) and on facial mimicry (Dimberg 1982; Mojzisch et al. 2006; Hess and Fischer 2013) predicts that observing others’ facial expressions may trigger an internal replication of the observed expression in the observer through the activation of areas involved in both the perception and execution of a given motor act. Recent theoretical perspectives further suggest that emotional expressions may recruit mirror-like mechanisms beyond the classical “motor/premotor” mirror neuron system for actions, supporting not only action understanding but also social synchronization and emotional attunement. Supporting this hypothesis, emotional facial expressions activate not only premotor regions but also emotional–interoceptive areas involved in the experience and expression of emotions, such as the anterior cingulate cortex (ACC) and the aforementioned AI (Caruana et al., 2020; Lombardi et al. 2023; Del Vecchio et al. 2024). However, the possible functional and structural relationships between these emotional–interoceptive regions and the motor/premotor ones have yet to be clarified.

Summing up, although various studies have investigated the specific contribution of selected frontal and perisylvian areas to the perception of emotional facial displays, as well as the possible existence of separate systems for the motor and emotional mirroring of others’ expressions, an unifying picture is still lacking to clarify whether all the areas involved in this process are reciprocally connected or mutually independent. In a first effort to shed light on this topic, in a recent study, we investigated it in epileptic patients using intracranial recordings, high-frequency electrical stimulation (HF-ES), and cortico-cortical evoked potentials (CCEPs) (Del Vecchio et al. 2024). Based on CCEPs results, we found that the frontal and perisylvian regions involved in this perceptual process can be grouped into two discrete clusters, each comprising areas with functional similarities. The first cluster is rooted in motor territories - constituted by the Rolandic operculum (RO) and a region between the frontal eye fields and the ventral premotor cortex, designated as frontal eye fields/ventral premotor cortex (FEF/vPMC), whose electrical stimulation evoke facial movements, including smiling expressions, as well as eyelid clonicity and contralateral aversive eye/neck movements. According to our hypothesis, in these territories the visual information related to others’ expressions is superimposed onto the observer’s own motor representations, thereby implementing a “motor resonance” of the observed facial display and building a spatial salience representation to support top-down attentional control. For simplicity, we refer to this set of areas as “the premotor cluster”. The second cluster encompasses emotional–interoceptive regions, such as ACC, AI, OFC, and the vmPFC as well as lateral prefrontal territories, such as the inferior frontal gyrus (IFG) and the middle frontal gyrus (MFG), known to encode contextual cues of the perceptual process. In this second cluster, the visual representation of others’ emotional displays may be encoded by activating the same visceromotor pathways that mediate affective responses in the individuals we observe, thereby conveying an “emotional resonance” between agent and observer in the service of social synchronization and emotional contagion (Cuccio and Caruana 2022). Based on the CCEP results, the premotor and the limbic/prefrontal clusters do not appear to be reciprocally interconnected (Del Vecchio et al. 2024). This suggests that the areas encompassing these clusters can act simultaneously during emotional face processing, but through independent mechanisms rooted in different networks, likely contributing to different aspects of emotional resonance (Caruana 2022; Cuccio and Caruana 2022). However, our previous study (Del Vecchio et al. 2024), while outlining the distinctive features of these clusters, has two limitations: first, it assessed effective connectivity using CCEPs obtained from the F-TRACT database, which did not allow for the creation of regions of interest (ROIs) precisely at the electrode locations; second, it did not identify the fiber bundles connecting the constituent regions of the various clusters.

The present study aims to establish the first multimodal connectivity architecture, combining tractography (DTI) and resting-state functional MRI (rsfMRI) connectivity methods, to overcome the lack of precise ROI-based connectivity and the absence of identified fiber bundles. Both these methods enabled connectivity analyses based on ROIs that precisely corresponded to the electrode sites identified by Del Vecchio and colleagues (Del Vecchio et al. 2024). In addition, while the tractography enabled the identification of fiber bundles connecting responsive regions, rsfMRI provided a complete, additional, picture of the functional network in which each studied territory is embedded.

DTI analyses reveal that these regions are organized into three majors anatomically and functionally coherent networks. Specifically, we identified two networks that largely replicate the functional clusters previously reported, alongside a third network that appears to act as an interface between them and as a source of visual input. Resting-state functional connectivity analyses revealed both shared and dissociable patterns of connectivity among these networks, partially corresponding with the canonical Default Mode, Ventral Attention, Dorsal Attention, Frontoparietal, and Sensorimotor networks. Overall, the convergence of structural and functional findings suggests that the frontal face-processing system is characterized by a distributed yet highly organized architecture.

Here we provide the first detailed anatomical and functional connectivity framework underlying the electrophysiological characteristics of the frontal and insular regions involved in emotional face perception. The networks identified here may represent the anatomical substrates for the hypothesized mechanisms through which ‘emotional resonance’ and ‘motor resonance’ operate - potentially in parallel - during facial expression recognition, as well as how these networks integrate contextual and attentional inputs from lateral prefrontal regions.

## Materials and methods

The structural connectivity was analyzed using diffusion tensor imaging (DTI), based on both deterministic and probabilistic approaches. The deterministic tractography was performed using the dataset of the DSI Studio software (http://dsi-studio.labsolver.org*)* and constituted by 1065 subjects. Since the deterministic algorithm implemented in DSI Studio is highly restrictive, it may not allow optimal reconstruction of certain white matter tracts, particularly those characterized by a high degree of fiber crossing. To address this limitation, we complemented this analysis with an additional probabilistic tractography (FSL software; https://fsl.fmrib.ox.ac.uk/fsl/docs/#/*)* performed on 12 healthy subjects from the University of Parma dataset and 4 subjects from the WU-Minn dataset of the Human Connectome Project (HCP; Van Essen et al., 2013), which has been successfully employed in previous studies (Gerbella et al. 2021; Di Cesare et al. 2021).

The functional connectivity was studied using resting-state functional magnetic resonance imaging (rsfMRI). The rsfMRI data were obtained from the Human Connectome Project (HCP) S1200 dataset, selecting 44 healthy subjects.

### Diffusion tensor imaging (DTI)

#### Scanning protocols for the probabilistic tractography

The data from 12 healthy humans (5 Males, 7 Females, range age 21–35, mean age 28) were acquired in a 3 T MR scanner (GE MR750) equipped with a dedicated 8 channel head-coil at Parma University Hospital.

The sequence protocol included a structural T1 weighted BRAVO sequence for anatomical reference, with TR/TE 57,501/3.96 ms, 0.5 × 0.5 × 0.9 mm3 voxel, and a diffusion spin echo single shot echo planar imaging (EPI) sequence, with TR/TE 56,586/82.4 ms, 2 × 2 × 2 mm3 isotropic voxel, 64 diffusion directions with an effective b value of 1000 s/mm2 and 8 images with an effective b value of 0 s/mm2 both in anterior–posterior phase encoding direction and in the reverse phase encoding direction.

Data from additional three subjects (2 Males and 2 Females, range age 26–35, mean age 30.5) from the WU-Minn data set of the Human Connectome Project (HCP; Van Essen et al. 2013) were included in our analysis, to provide a preliminary consistency check whether the results obtained from our dataset were replicable also in data with smaller voxel size. These subjects (numbers 102816,102311,128935, and 100610) have been acquired with a 3 T MR scanner (Siemens) and the sequence protocol included a structural T1 weighted BRAVO sequence for anatomical reference, with TR/TE 2400/2.14 ms, 0.6999 × 0.6999 × 0.6999 mm^3^ voxel, and a diffusion spin-echo single shot EPI sequence, with TR/TE 5520/89.5 ms, 1.25 × 1.25 × 1.25 mm3 isotropic voxel, 96 diffusion directions with an effective b value of 3010 s/mm^2^ 90 diffusion directions for each shell defined with b-values of 1000, 2000, and 3000 s/mm2, plus 18 images with an effective b value of 0 s/mm^2^, scanned along two phase encoding directions (L/R and R/L).

#### Deterministic tractography

Concerning the analysis performed with DSI Studio, this software employs the deterministic fiber tracking method and provides functionalities encompass reconstruction, deterministic fiber tracking (utilizing the TEND algorithm), and 3D visualization (Christidi et al. 2016).

Spherical regions of interest (ROIs) with a 5 mm radius were created using the MarsBaR toolbox for the SPM software (see below for details) and subsequently placed within the DSI Studio interface (http://dsi-studio.labsolver.org), employing the ICBM152_adult MNI template (Schilling et al. 2019) to identify fiber bundles passing between ROI pairs in the frontal lobe. The diffusion data were reconstructed in the MNI space employing q-space diffeomorphic reconstruction (Yeh and Tseng 2011) to obtain the spin distribution function (Yeh et al. 2010). A diffusion sampling length ratio of 1.7 was utilized, and the output resolution is 2 mm isotropic. Restricted diffusion was quantified using restricted diffusion imaging (Yeh et al. 2016). A deterministic fiber tracking algorithm (Yeh et al. 2013) was applied, incorporating augmented tracking strategies (Yeh 2020), aimed at enhancing reproducibility.

The tractography was generated using the following tracking parameters: the anisotropy threshold was randomly selected; the angular threshold was set at 60 degrees, with a step size of 1.20 mm. Tracks with length shorter than 30 or longer than 200 mm were discarded. A total of 10,000 tracts were calculated (*https://journals.plos.org/plosone/article? id=*10.1371/journal.pone.0080713*).* To confirm that by modifying the tractography parameters the results did not substantially change, we generated tractography also using different angular thresholds, ranging from 45° to 65°, different step sizes, ranging from 0.5 to 1.2 mm, and length cut offs, ranging from 20 to 300 mm.

#### Probabilistic tractography

Concerning the Parma database and the WU-Minn data set, the diffusion images were analyzed with the FMRIB Software Library (FSL) tools (version 5.0.9) (Glasser et al. 2013; Jenkinson et al. 2012; Smith et al. 2004; Woolrich et al., 2009). First, employing the reversed phase data, raw images were corrected for head motion and distortions induced by eddy currents and difference in the susceptibility distribution within the brain using the FSL’s tools TOPUP (Andersson et al. 2003; Smith et al. 2004) and EDDY (Andersson and Sotiropoulos 2016). Then, diffusion tensor estimate was carried out with FSL’s BEDPOSTX tool, which enables to model multiple fiber crossing within each voxel of the brain (Behrens et al., 2003; Behrens et al. 2007).

Subsequently, a probabilistic tractography analysis was executed with FSL’s PROBTRACKX tool (using curvature threshold = 0.2, number of samples = 5000) (Behrens et al., 2003; Behrens et al. 2007). For each tract, the streamlines were seeded from two mask images, constituted by spherical region of interest (ROI), testing the structural connection from seed A to seed B and vice versa (from B to A): the final output is the sum of the connectivity distributions resulting from the two analyses.

To compare and average the resulting tracts, the assessment was performed in the Montreal Neurological Institute 152 (MNI 152) space (Grabner et al. 2006). Each voxel of the resulting images contained the value of the total number of streamlines crossing in voxel, producing maps of connectivity distribution showing the most probable pathway between the selected seeds: to reduce false negative and false positive rate and obtain the best tractography reflection of tracers in monkey studies (Azadbakht et al., 2015), a threshold of 10% on the maximum number of streamlines reported by PROBTRACKX was chosen.

Finally, to assess the repeatability of the identified tracts across subjects, we first performed a subject-by-subject inspection and excluded those exhibiting anatomically implausible tracts. This step reduced potential sources of false positives and consisted of removing tracts unlikely to be anatomically meaningful. We then retained only tracts showing anatomically plausible pathways in at least 60% of the subjects, thereby discarding those not identified in more than half of the sample. Subsequently, each retained tract was binarized, and the binarized tracts were summed and averaged across subjects. The resulting value is proportional to the number of voxels in which streamlines are present in at least 60% of the examined subjects.

#### Identification of the white matter tracts

In the analysis carried out using DSI Studio, the identity of the white matter tracts found was assessed, firstly, using the function *Recognize and Cluster* and, subsequently, matching them with the HCP842_tractrography atlas.

Concerning the FSL based analysis, to identify at which fasciculi belonging the white matter tracts observed, we overlap our results with the white matter tracts (Rojkova et al. 2016) of the atlas present in the Tractotron tool in BCBToolkit software (http://toolkit.bcblab.com/; Foulon et al., 2018). A threshold of 10% on the maximum number of streamlines was used also for the tracts of the BCBTToolkit.

### Functional resting-state imaging

#### Data acquisition

Functional connectivity of rsfMRI data of 44 healthy unrelated adults (age: 22–35, 14 males) from the Human Connectome Project (HCP) S1200 release of resting state fMRI (Van Essen et al., 2013) were used for this study. Data included 1200 frames of multiband, gradient-echo planar imaging (Smith et al., 2013) acquired in approximately 15 min with the following parameters: echo time, 33.1 ms; flip angle, 52°; field of view, 280 180 mm; matrix, 140 90; and voxel dimensions, 2 mm isotropic resolution; TR, 0.72 s (Ugurbil et al., 2013).

The study used the HCP Young Adult test–retest subsample (*N* = 44, 30 females, 14 males) to ensure methodological homogeneity and data quality. An a priori power analysis (G*Power 3.1.9.7; one-sample t-test, one-tailed, α = 0.05, power = 0.99) indicated that 21 participants are required to detect a large effect (Cohen’s d = 0.9). Our analyses largely exceed this requirement, also considering the gender balance, as they were conducted on 44 subjects.

#### Preprocessing and analysis of imaging data

The resting state fMRI data were preprocessed using Statistical Parametric Mapping 12 (SPM12) software (https://www.fil.ion.ucl.ac.uk/spm/*).* It was created a pipeline including the following steps: slice timing correction, rigid-body correction for head motion, co-registration of the fMRI volumes with the high-resolution T1-weighted structural image, cortical segmentations of the T1 image, spatial normalization of functional volumes and functional smoothing with a Gaussian kernel of 6 mm3 full-width half-maximum. Structural segmentation and spatial normalization allowed it to match the human template brain.

A seed-based resting-state functional connectivity analysis was conducted to assess the correlation between the BOLD (Blood Oxygen Level Dependent) time series of each seed/ROI and those of all other voxels across the brain. The result is a connectivity map showing Z-scores for each voxel indicating how well its time series correlates with the time series of the ROI. This analysis was executed using the Functional Connectivity (CONN) toolbox (https://www.nitrc.org/projects/conn*)*, a MATLAB/SPM-based cross-platform open-source software. After preprocessing, images were band-pass filtered to 0.008–0.09 Hz and motion regressed to diminish the impact of noise. Normality was achieved thanks to a Fisher Z transformation applied to correlation maps. A general linear model was used to calculate the ROI-based functional connectivity of each subject (1st level analysis), determining single-subject, functional networks.

First-level results of ROI-to-voxel functional connectivity maps were then included into a second-level general linear model, obtaining population-level analysis. When examining the results of a second-level analysis, the CONN toolbox identifies significant voxels using a combination of height (voxel-level) and extent (cluster-level) thresholds. The height threshold defines the minimum statistical intensity a voxel must reach to be included in a cluster (*p* < 0.001, uncorrected), whereas the extent threshold defines the minimum cluster size (i.e., number of contiguous voxels) required for the cluster to be considered significant (FDR < 0.05). In two cases (pACC and sACC), in which the signal was particularly low, the threshold was adjusted using an uncorrected p-value height threshold of *p* < 0.01. Only sectors with positive correlations were included in second-level analysis. Independent t-tests were done to detect the voxels with higher connectivity.

To determine the extent to which the functional networks observed here overlap with the classical Yeo networks (Yeo et al., 2011), we performed a quantitative analysis. This analysis allowed us to assess the number of voxels in each of our functional networks that belong to the different Yeo networks. Specifically, we calculated the ratio between the number of voxels in our functional networks that overlap with the Yeo networks and the total number of voxels in each of our networks.

To determine the extent to which the functional networks observed here overlap with each other, we calculated the Dice coefficients, using the number of the voxels of each functional network as factors. In other words, the Dice coefficients calculated, ranging from 0 to 1, quantify the overlap between different functional networks, with voxel counts in each network used as the factors in the calculation.

### ROIs’ location

**Fig. 1:**
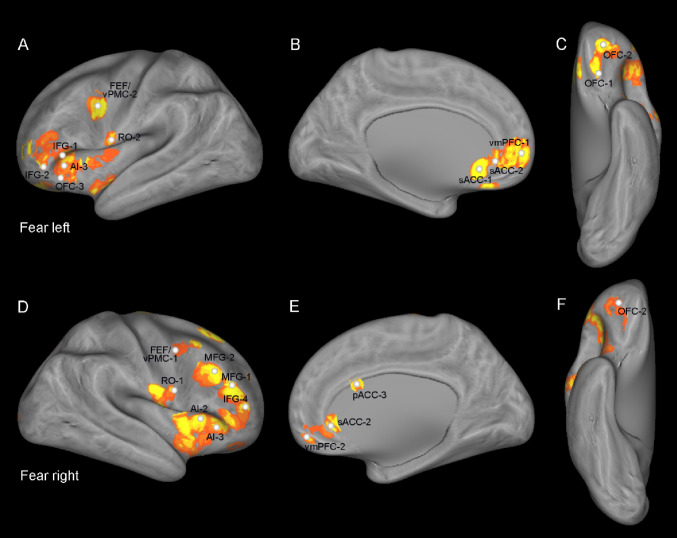
Fear condition. Location of the ROIs, represented by white spheres, based on the density maps used in Del Vecchio et al. (2024) showing significant differences between the iERPs elicited by the observation of fear and neutral expressions. The color scale indicates the percentage of responsive sites within a disk 1 cm in radius and centered on each node of the mesh. Abbreviations: *AI*, anterior insula; *IFG*, inferior frontal gyrus; *MFG*, medial frontal gyrus; *OFC*, orbitofrontal cortex; *pACC*, pregenual cingulate cortex; *sACC*, subgenual cingulate cortex; *RO*, rolandic operculum; *FEF/vPMC*, frontal eye fields/ventral premotor cortex; *vmPMF*, ventro-medial prefrontal cortex

**Fig. 2:**
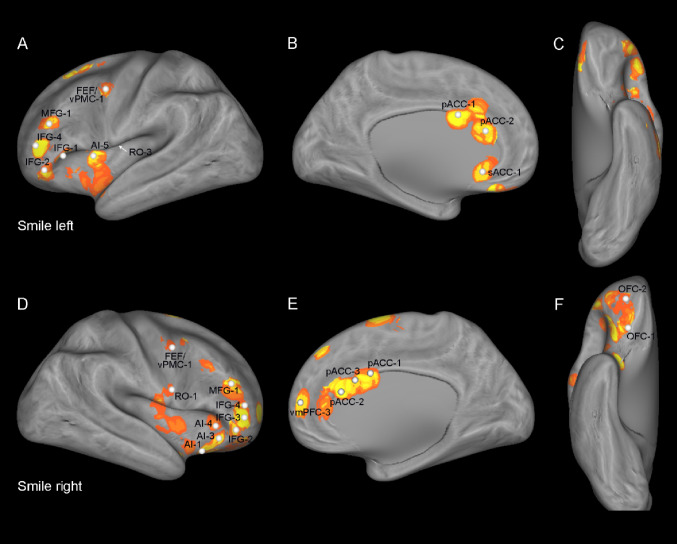
Smile condition. Location of the ROIs, represented by white spheres, based on the density maps used in Del Vecchio et al. (2024) showing significant differences between the iERPs elicited by the observation of smile and neutral expressions. The color scale indicates the percentage of responsive sites within a disk 1 cm in radius and centered on each node of the mesh. Abbreviations as in Fig. 1

The ROIs used for both tractography and functional connectivity analysis included the following frontal territories: the ventromedial prefrontal cortex (vmPFC), the anterior cingulate cortex (sACC, pACC), the orbitofrontal cortex (OFC), the pars triangularis of the inferior frontal gyrus (IFG), the anterior insular cortex (AI), the Rolandic operculum (RO) and the FEF/vPMC (FEF/vPMC). These subsectors were chosen based on the locations in which the iERP responses of Del Vecchio and colleagues (2024) showed greater activity to the observation of emotional expressions versus neutral ones. Specifically, we, first, identified the sites where either fear or smile expressions elicited a significant iERP modulation relative to neutral expression (Figs. 1 and 2; Table 1 in Del Vecchio et al. 2024). Subsequently, we computed continuous maps of the results to provide a continuous view of the topographic pattern of active leads, i.e., the proportion of leads significantly selective to emotional expressions out of the overall number of responding leads. To obtain these maps, in a previous study (Del Vecchio et al. 2024), we built a circular mask based on the geodesic distance between two cortical points (i.e. the minimum pathway within the grey matter connecting the source and the target nodes). For each cortical node, we defined the nodes within a 1-cm geodesic distance from the original node and weighted the contribution of each node by a sigmoid function. Node weight was defined as a logistic function with unitary amplitude, a steepness of 2 and a midpoint at 7.5 mm. As a result, each node of the cortical mesh was associated with a collection of surrounding nodes, with all nodes within 5 mm of the origin maximally weighted, whereas those between 5 and 10 mm were gradually reduced in weight, to avoid edge effects. Then, we computed the the proportion of leads significantly selective to emotional expressions with respect to the overall number of responding leads. These maps provide a topographic picture of the frontal/insular selectivity of the emotional expressions. These maps, thresholded at 10% to exclude the contribution of sparsely responsive regions, provided a topographic picture of the frontal/insular selectivity of the emotional expressions. We built the ROIs for the DTI investigation, defining their center of gravity as the sector of the region with a high density of responsive sites. Since each cortical sector was characterized by multiple peaks of density, in each territory different centers of gravity, and corresponding ROIs were defined. For example, four ROIs in the AI and three in the pACC were built (Figs. 1 and 2; Table 1).

In the case of rsfMRI, to obtain a more comprehensive view of the resting-state functional connectivity of each studied territory, the ROIs were positioned at the center of each territory by averaging the coordinates of the ROIs used in the DTI analysis. Thus, the ROIs used in the rs-fMRI study can be defined as the centers of gravity of the ROIs employed for each territory in the tractography analysis. (Table 2).

For both analyses, ROIs were created using the MARSBAR toolbox in the SPM software and consisted of spheres with a 5 mm radius. However, due to the 2D representation of the brain in the Caret template, there were instances where the projected ROIs did not fully encompass the white matter or were not entirely situated within the grey matter. To address these issues, some adjustments can be made to the ROI coordinates.

**Table 1:** Coordinates of ROIs based on the significant responses of Del Vecchio et al. (2024) used in human probabilistic tractography studies and given in MNI standard coordinates

	Rois	x	y	z	SMILE (L)	SMILE (*R*)	FEAR (L)	FEAR (*R*)
pACC	pACC_1	−4	6	30	•			
	pACC_2	−4	23	20	•			
	pACC_3	4	15	26				•
sACC	sACC_1	−4	23	−6	•		•	
	sACC_2	± 4	39	−2			•	•
vmPFC	vmPFC_1	−10	55	4			•	
	vmPFC_2	7	51	−10		•		
	vmPFC_3	7	53	13				•
IFG	IFG_1	−53	34	0	•		•	
	IFG_2	± 47	37	−7	•	•	•	
	IFG_3	41	40	3		•		
	IFG_4	± 37	41	9	•	•		•
AI	AI_1	26	17	−14		•		
	AI_2	30	18	8				•
	AI_3	± 31	30	4		•	•	•
	AI_4	−34	6	7	•			
OFC	OFC_1	± 19	21	−25		•	•	
	OFC_2	± 23	43	−13		•	•	•
	OFC_3	± 40	26	−10		•	•	
RO	RO_1	61	3	19		•		
	RO_2	−59	−2	10			•	
	RO_3	−51	−4	6	•			
FEF/vPMC	FEF/vPMC_1	± 46	2	43	•	•		
	FEF/vPMC_2	−41	2	32			•	
MFG	MFG_1	± 46	33	23	•			•
	MFG_2	41	22	30				•

Dots represent the presence of a tractography-derived analysis for that seed in the given condition (smile or fear) and hemisphere (L = left, R = right)

**Table 2:** Coordinates of seeds used in human resting state functional connectivity studies and given in MNI standard coordinates

Rois	x	y	z
vmPFC	−13	50	−2
sACC	−4	23	−5
pACC	−5	24	26
IFGptpt	−48	36	0
AI	−38	18	−7
RO	−59	−2	5
FEF/vPMC	−43	2	39

## Results

### Diffusion tensor imaging (DTI)

#### Deterministic analysis

The following results have been obtained by using the deterministic approach (DSI Studio) on the ICBM152_adult template. Overall, the deterministic analysis revealed that the connections between the frontal and insular ROIs encoding emotional expressions in both hemispheres, and regardless of its positive or negative valence, include the inferior frontal occipital fasciculus (IFOF), the arcuate fasciculus (AF), the anterior commissure bundle (AC), the cingulum bundle (CB), the uncinate fasciculus (UF), and the superior longitudinal fasciculus (SLF). Below we describe in detail all the features that have been identified by analyzing the pairwise connections between the frontal regions involved in the visual processing of emotional expressions.

**Table 3:** Fasciculi connecting the various ROIs

ROIs	OFC	vmPFC	MFG	AI	FEF/ vPMC	IFG	sACC	pACC	RO
OFC		UF		UF/IFOF			AC		
vmPFC	UF						AC/CB	CB	
MFG					AF				SLF
AI	UF/IFOF					IFOF/AF			
FEF/vPMC			AF			AF/SLF			FAT
IFG				IFOF/AF	AF/SLF				
sACC	AC	AC/CB						CB	
pACC		CB					CB		
RO			SLF		FAT				

**Fig. 3:**
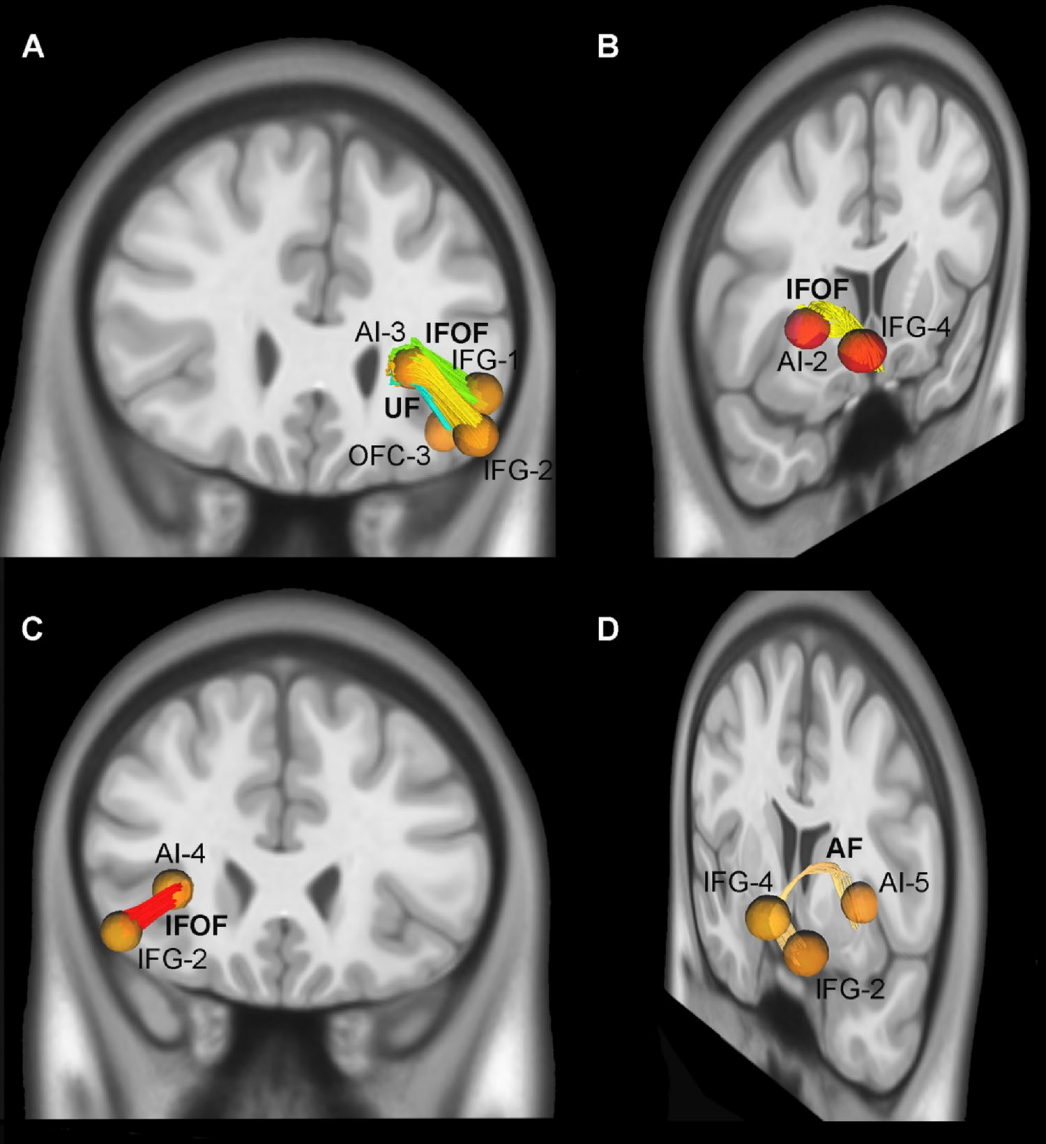
Exemplificative white matter tracts connect AI, IFG and OFC via the uncinate fasciculus (UF), the inferior longitudinal fasciculus (IFOF), and the arcuate fasciculus (AF)

**Fig. 4:**
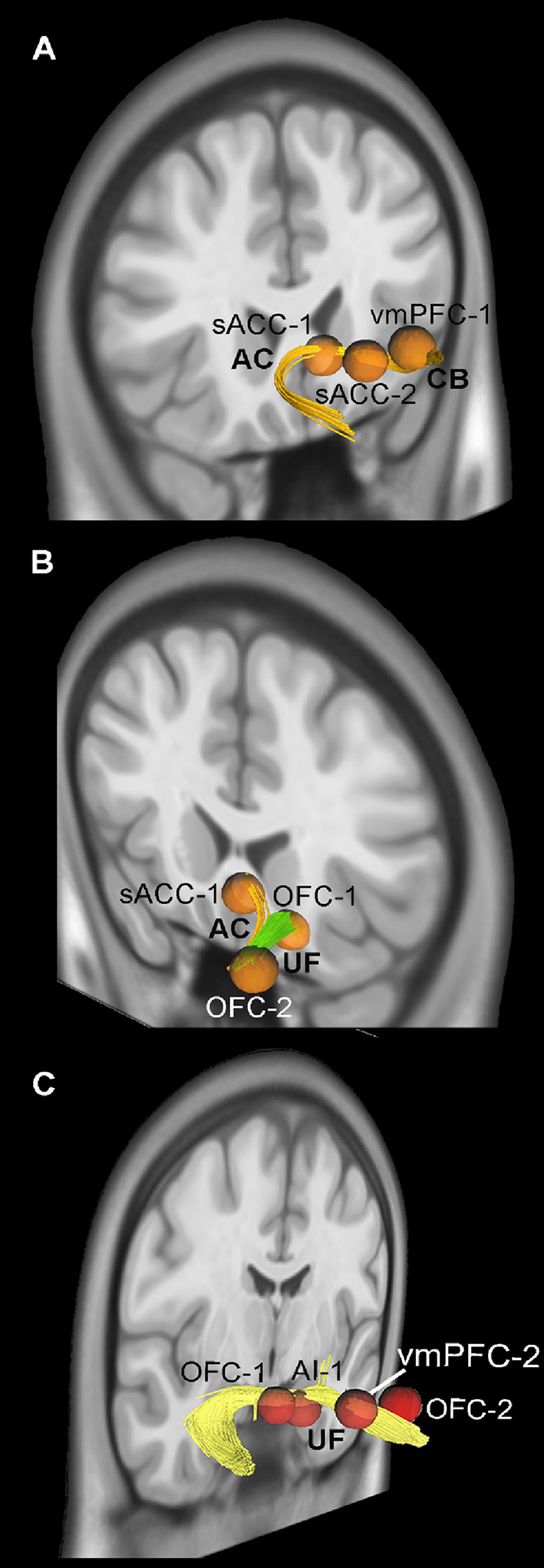
In A, exemplificative white matter tracts connect sACC, and vmPFC via the cingulate bundle (CB) and the anterior commissure (AC). In B and C exemplificative white matter tracts connect OFC, sACC, and vmPFC via uncinate fasciculus (UF), and anterior commissure (AC)

**Fig. 5:**
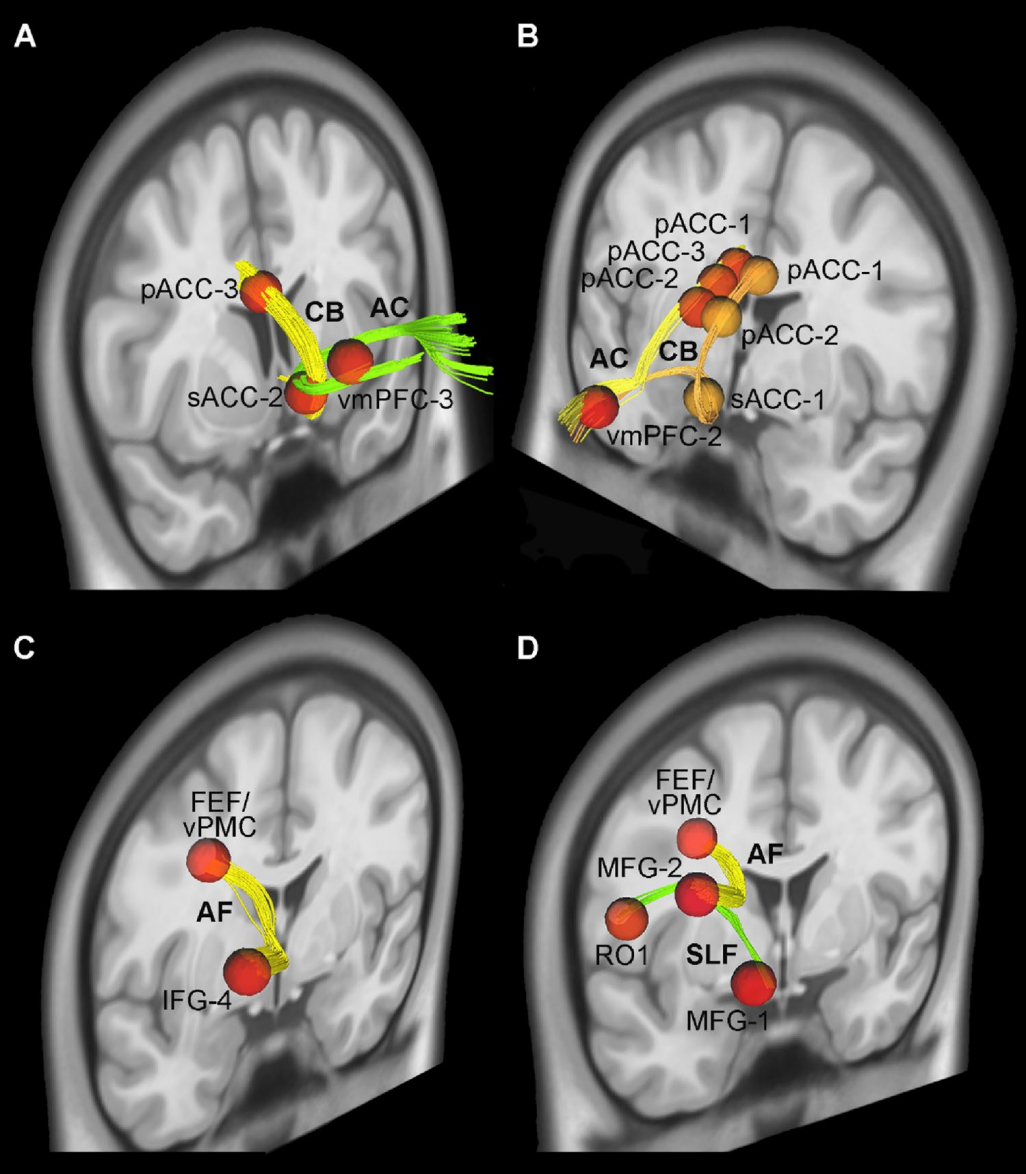
In A and B, exemplificative white matter tracts connect pACC, sACC, and vmPFC via the cingulate bundle (CB), anterior commissure (AC). In C and D exemplificative white matter tracts connect FEF/vPMC, RO, IFG, and MFG via arcuate fasciculus (AF), and superior longitudinal fasciculus (SLF)

In particular, considering both hemisphere and ROIs from both conditions we found that: (1) the IFOF and the AF connects the AI with the IFG (Fig. 3A, B and C, and 3D; Table 3); (2) the AC connects the sACC with the vmPFC (Figs. 4A and B and 5A, and 5B; Table 3); (3) the UF connect the OFC with the AI and vmPFC (Figs. 3A and 4B, and 4C; Table 3); (4) the CB connects all the cingulate territories to one another and the sACC with the vmPFC (Fig. 5A and B; Table 3); (5) the AF connects IFG with FEF/vPMC (Figs. 5C; Table 3); (6) the SLF connects the vPMC and RO with MFG (Fig. 5D; Table 3). The data from individual condition and hemisphere are described in detail in the supplementary material.

**Fig. 6:**
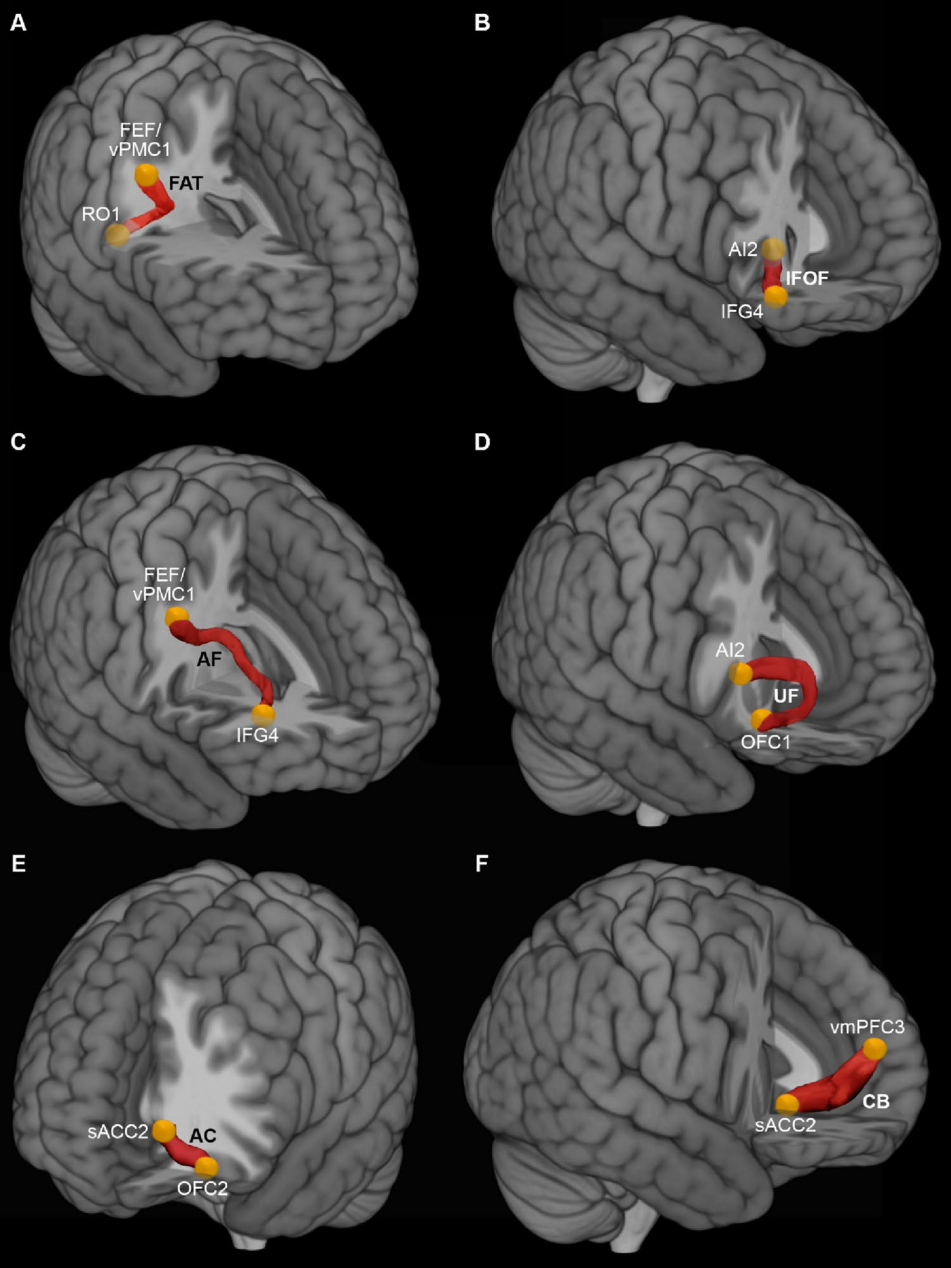
Averaged white matter tracts connecting RO with FEF/vPMC (**A**), AI with IFG (**B**), FEF/vPMC with IFG (**C**), AI with OFC (**D**), sACC with OFC (**E**), and vmPFC with sACC (**F**) showed in lateral views of 3D reconstructions

#### Probabilistic analysis

The Ball-and-stick probabilistic analysis was performed using FSL on 12 subjects from our database and 4 subjects from the WU–Minn dataset of the HCP. All results obtained with the deterministic approach were largely confirmed in both datasets using this type of analysis as well (Fig. 6 and Supplementary Tables 5 and 6). Specifically, for each tract, the connection between at least one pair of ROIs was observed in all or almost all subjects considered (see Supplementary material). In addition to the results observed in the deterministic tractography, the probabilistic approach showed that, in the large majority of subjects of both our and WU–Minn datasets, the IFOF connects the OFC with AI, and the frontal aslant tract (FAT) connects RO with FEF/vPMC (Fig. 6 and Supplementary Tables 5 and 6). The Supplementary Tables 5 and6 also report the average number of streamlines for each tract, showing strong consistency for almost all the pairs of ROIs considered.

#### Summary of the tractography results

Our tractography results show that the CB and UF connect regions involved in the emotional cluster defined by Del Vecchio et al. (2024), namely the OFC, AI, IFG, and mesial and cingulate prefrontal regions (pACC, sACC, and vmPFC). Specifically, the mesial prefrontal regions (pACC, sACC, and vmPFC) are interconnected through the CB; the OFC and AI are interconnected via the UF and the IFOF, which also links them to the IFG; the OFC and vmPFC are connected through the UF; and the OFC is connected with the sACC through the AC. A distinct system involves the premotor regions (RO and FEF/vPMC), which are reciprocally interconnected through the FAT. Finally, the SLF and AF connect the FEF/vPMC with the IFG, while only the SLF links the MFG with the vPMC and RO.

### Functional resting state analysis (rsfMRI)

**Fig. 7:**
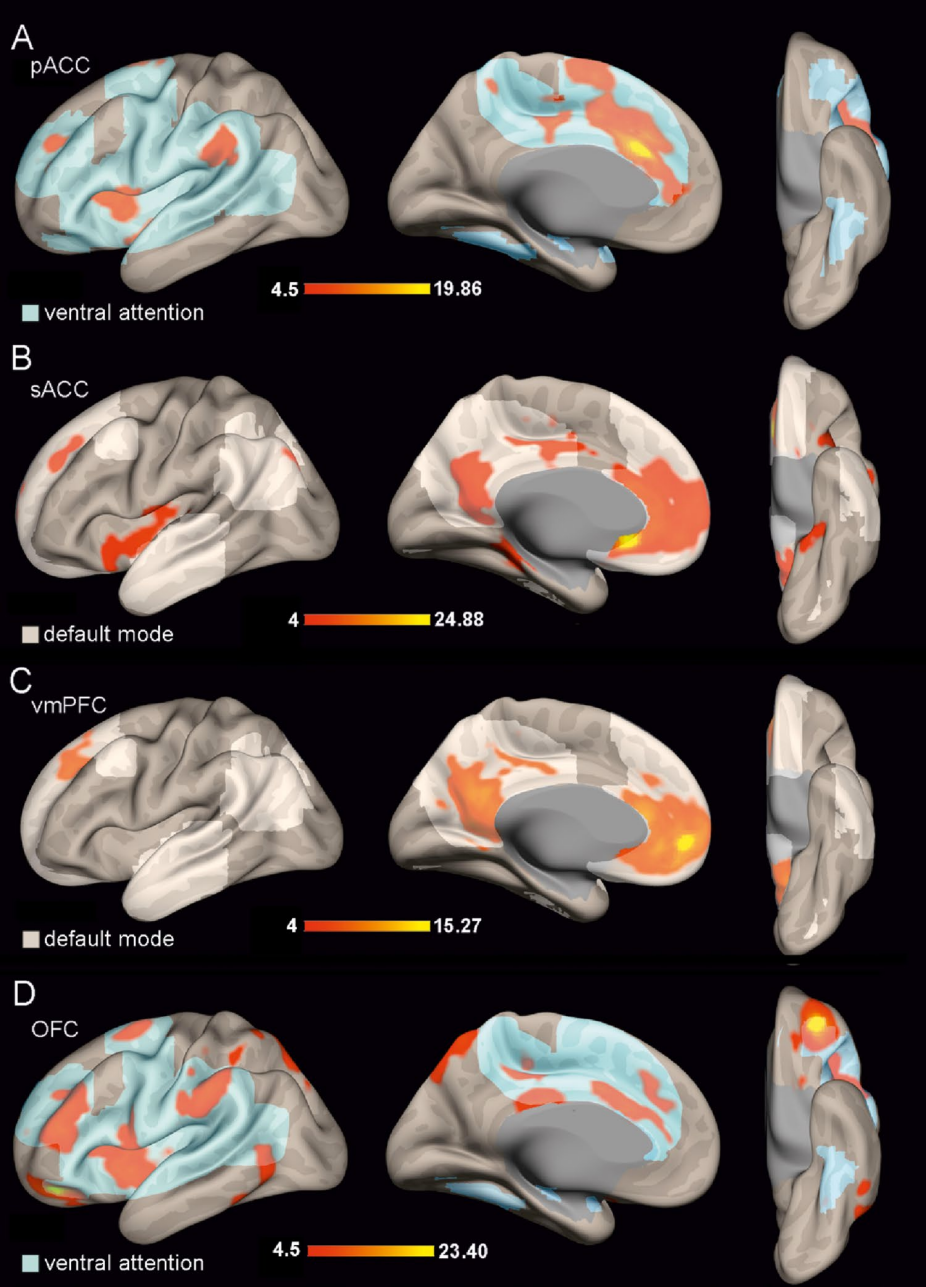
The functional network of pACC (**A**), sACC (**B**), vmPFC (**C**), and OFC (**D**) are shown in lateral, mesial, and orbital views of 3D brain reconstructions. The color bar below each functional network represents the range of the t-value. The shaded areas are a smoother representation of the functional network of Yeo (Yeo et al. 2011) with which our functional network mainly overlaps

**Fig. 8:**
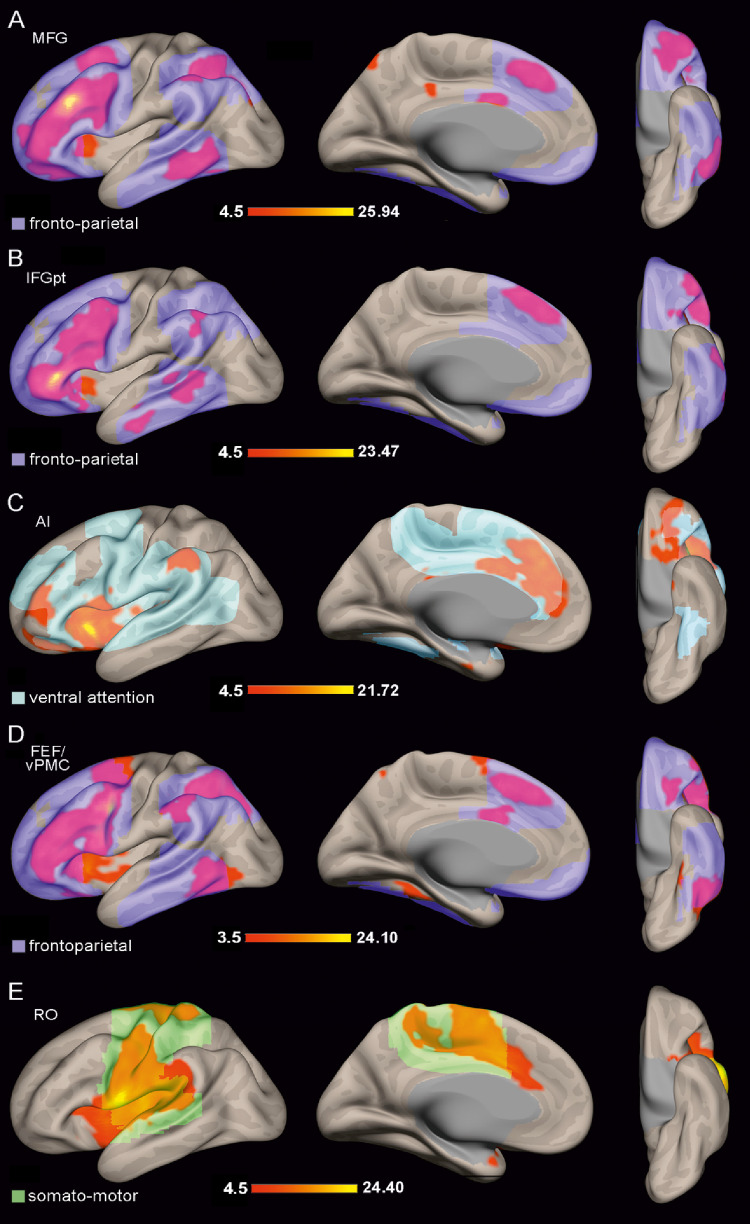
The functional network of MFG (**A**); of IFG (**B**); of AI (**C**); of FEF/vPMC (**D**); of RO (**E**) are shown in lateral, mesial, and orbital views of 3D brain reconstructions. The color bar below each functional network represents the range of the t-value. The shaded areas are a smoother representation of the functional network of Yeo (Yeo et al. 2011) with which our functional network mainly overlaps

**Fig. 9:**
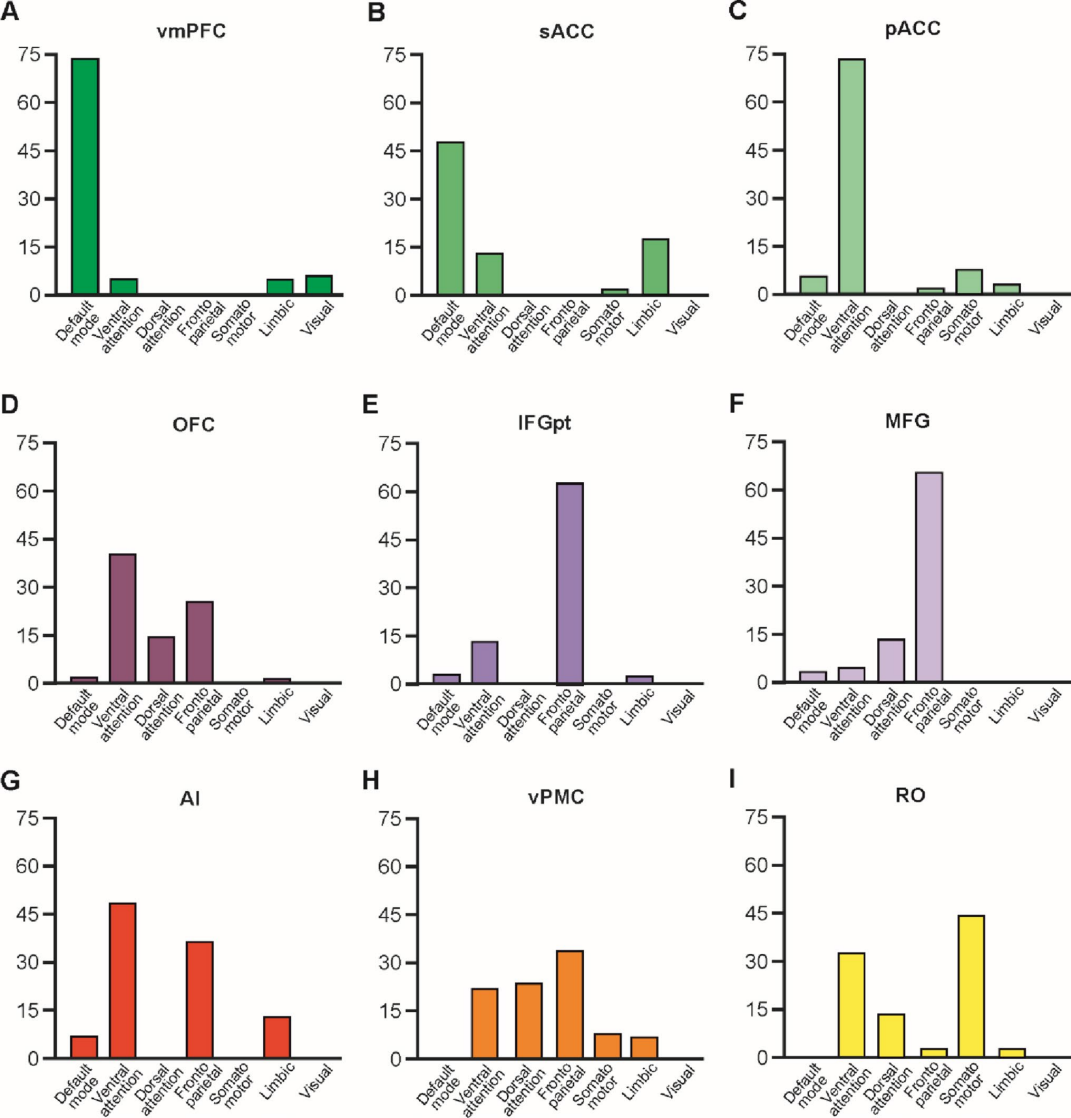
Percentage of the voxels overlapping between the functional network here identified and those described by Yeo (Yeo et a. 2011)

As functional connectivity is widely acknowledged to be constrained by underlying anatomical connectivity (Behrens and Sporns 2012; Howells et al. 2020), we sought to gain a broader perspective on the contribution of each region involved in emotional face processing. To this end, we conducted a resting-state functional connectivity analysis to characterize the functional networks associated with each region of interest (ROI) implicated in emotional face coding (Del Vecchio et al. 2024). Only left-hemisphere results are reported, as the right hemisphere exhibited a highly similar activation pattern. The ROI-to-voxel connectivity of each region is shown in Figs. 7 and 8. To provide a clearer description of the connectivity patterns obtained, Fig. 9 shows the percentage of voxels within the functional network identified for each ROI that belong into the ‘classical’ seven functional networks defined by Yeo (Yeo et al., 2011); in addition, Supplementary Fig. 1 shows the Dice coefficients obtained between the functional networks of the studied regions (for the detail see Materials and Methods).

The pACC is functionally connected with adjacent sectors of the cingulate gyrus (sACC and MCC), the AI, and the superior frontal gyrus (Figs. 7A). Considering the regions to which it is connected, it appears to be primarily involved in the ventral attention network (Fig. 9).

The sACC is functionally coupled primarily with adjacent sectors of the vmPFC and the AI, in addition activated voxels have been found in the precuneus and the superior frontal gyrus (Figs. 7B). Considering altogether the regions to which it is functionally coupled, it appears to be primarily involved in the default mode network (Fig. 9).

The vmPFC showed activation primarily in adjacent regions, including the sACC and the precuneus, both of which are part of the default mode network, the only Yeo network consistently connected to this ROI (Figs. 7C and 9). Accordingly, the Dice coefficients showed a strong functional coupling between the functional networks of vmPFC and sACC (Supplementary Fig. 1). Additional activation was observed in the superior frontal gyrus (Fig. 7C).

The OFC showed functional activated voxels also in the pACC and MCC as well as in the MFG, posterior STS, and, unexpectedly, the inferior parietal lobule (Fig. 7D and Supplementary Fig. 1). Based on this connectivity pattern, the OFC appears to be primarily connected with regions belonging to the ventral attention network (Fig. 9). Accordingly, the Dice coefficients showed a strong functional coupling between the functional networks of OFC and AI (Supplementary Fig. 1).

The MFG showed functionally activated voxels extending into the adjacent IFG and FEF/vPMC, whose functional networks were the most similar to that of the MFG according to the Dice coefficients (Supplementary Fig. 1). Additional activated voxels were observed in the superior and mesial frontal gyri, the AI, the OFC, the posterior inferotemporal cortex, and the superior parietal lobule (Figs. 8A). Among the Yeo functional networks, the MFG network overlapped predominantly with the frontoparietal network (Figs. 8A and 9).

Very similar to the MFG network is the IFG one, as shown by the Dice coefficients, and by a distribution of activated voxels into the adjacent IFG and MFG, the superior and mesial frontal gyri, the AI, the OFC, and the posterior inferotemporal cortex. Accordingly, the IFG network overlapped predominantly with the frontoparietal network (Figs. 8B and 9).

The AI showed coupled voxels in the pACC, the OFC, the mesial frontal gyrus, and in the IPL (Fig. 8C). The AI functional network overlaps especially with the ventral attention network of Yeo (Yeo et al. 2011) and the pACC network (Fig. 9 and Supplementary Fig. 1).

The FEF/vPMC network overlaps predominantly with the frontoparietal network described by Yeo et al. (2011), involving the MFG, IFG, AI, superior and medial frontal gyri, pACC, and IPL. In addition, a high percentage of voxels was observed in both the dorsal and ventral attention networks (Figs. 8D and 9).

The RO network primarily involved regions belonging to the somatomotor network as defined by Yeo et al. (2011), although the activated voxels also extended into territories associated with the ventral and dorsal attention networks (Figs. 8E and 9). Accordingly, the Dice coefficients indicated that the functional network of the RO overlaps especially with other territories in which movements can be evoked, such as FEF/vPMC, pACC, and AI (Supplementary Fig. 1).

In summary, the functional networks of the region of the “emotional” cluster defined in Del Vecchio and colleagues (Del Vecchio et al. 2024) primarily overlapped with the default mode network in the case of the sACC and vmPFC, with the ventral attention network for the pACC, AI, and OFC, and with the frontoparietal network for the IFG and MFG. In contrast, the functional networks of the territories of the “motor” cluster uniquely showed robust overlap with the dorsal attention network, in addition to the ventral attention network. Within this cluster, the RO exhibited a strong overlap with the somatomotor network, whereas the FEF/vPMC overlapped predominantly with the frontoparietal network.

## Discussion

In this study, we investigated the connectivity of the frontal and insular regions involved in the processing of emotional expressions by adopting a multimodal approach. First, we identified the ROIs based on our previous intracranial study (Del Vecchio et al. 2024), in which emotional expressions (smiling or fearful) were shown to significantly modulate iERPs compared to neutral faces. Next, we examined their intrinsic structural connectivity using both deterministic and probabilistic tractography and then, we computed their functional connectivity.

The regions encoding emotional facial displays and in which we identified the ROIs include: pregenual and subgenual portions of the anterior cingulate cortex (pACC and sACC), orbitofrontal cortex (OFC), anterior insula (AI), inferior and middle frontal gyri (IFG and MFG), and - at the level of the premotor cortex - the rolandic operculum (RO) and a regions between the frontal eye fields and the ventral premotor cortex (FEF/vPMC).

**Fig. 10:**
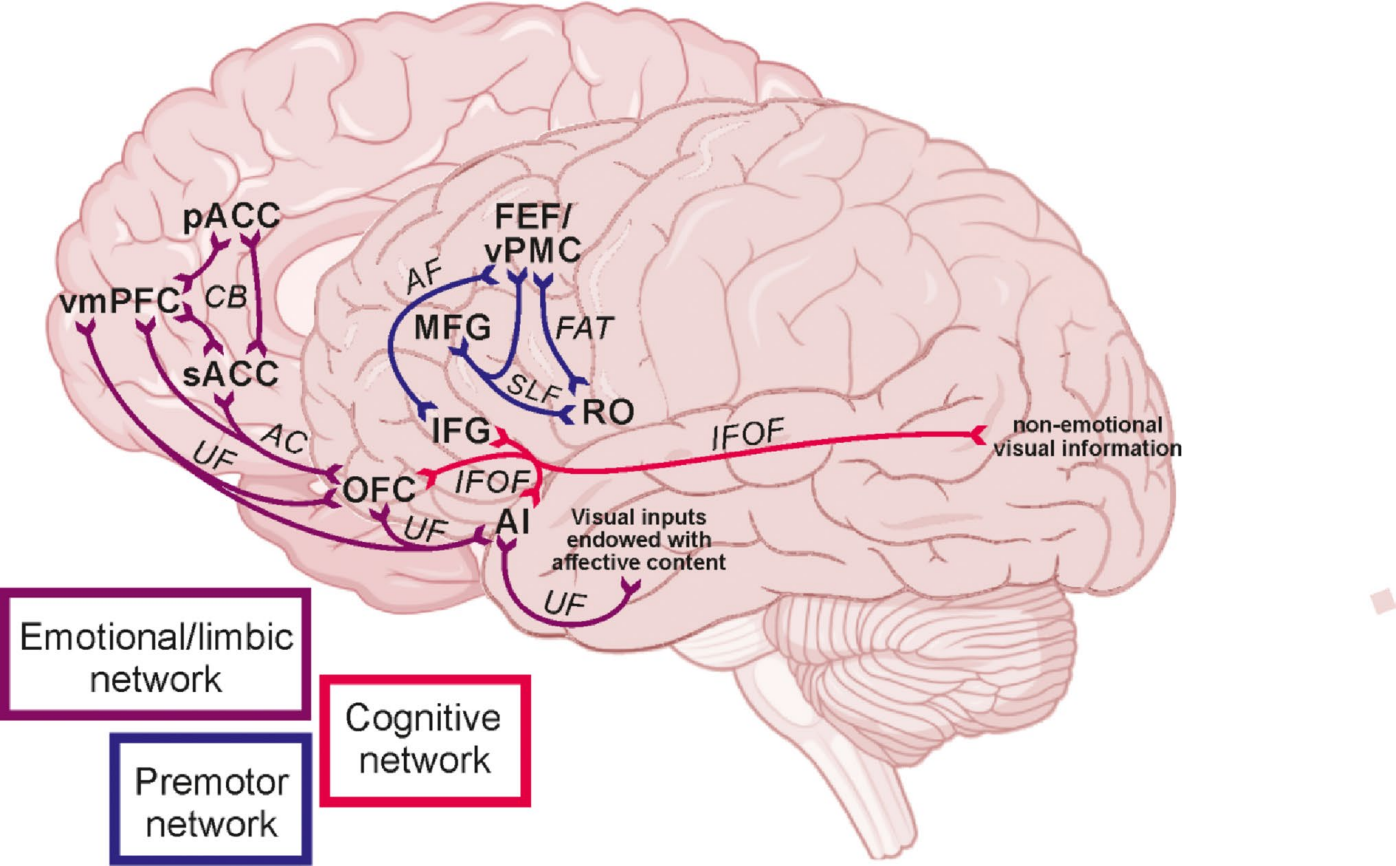
Summary of the anatomical connections of the three networks here identified

The tractography results indicate that intrinsic connectivity between these regions is supported by a limited number of tracts, which appear to form mainly internal loops within two clusters - a limbic/prefrontal and a premotor cluster - that we previously identified in functional terms in our earlier study (Del Vecchio et al. 2024). In particular, a system consisting of the cingulum bundle (CB), the uncinate fasciculus (UF), and the anterior commissure (AC) connects four regions of the aforementioned limbic/prefrontal cluster (Fig. 10). These areas - namely, the mesial prefrontal regions (pACC, sACC, and vmPFC), the orbitofrontal cortex (OFC), and the anterior insula (AI) - are all involved in emotional control, socio-emotional responses, and interoception, and may provide an anatomical substrate potentially relevant for processes such as emotional contagion. We named this set of areas “emotional network”.

A relatively distinct system involves a set of regions exactly corresponding to those of the premotor cluster of our previous study (Del Vecchio et al. 2024), we designated it as “premotor network”. The regions of this network - namely the RO and FEF/vPMC - are reciprocally interconnected through the frontal aslant tract (FAT; Fig. 10), a bundle typically associated with many visuo-motor functions, including orofacial movements and the production of voluntary laughter behavior (Martino et al. 2012; Chernoff et al. 2019; Gerbella et al. 2021). These regions are well known as engaged in the recognition of facial expressions but lack direct connections with temporal areas. Consequently, their recruitment during face processing is presumed to be mediated by other frontal regions. In particular, this premotor network may receive inputs potentially related to visual or audiovisual processing through two lateral prefrontal regions, the pars triangularis of inferior frontal gyrus (IFG) and the middle frontal gyrus (MFG), via the arcuate fasciculus (AF) and the superior longitudinal fasciculus (SLF). In turn, the IFG may receive purely visual input from the occipito-temporal cortices via the inferior fronto-occipital fasciculus (IFOF), whereas the MFG may receive audiovisual information conveyed by the arcuate fasciculus (AF) (Fig. 10). Thus, they represent relatively distinct territories within the limbic/prefrontal cluster described by Del Vecchio and colleagues (Del Vecchio et al. 2024), and were therefore considered as a distinct network, here designated as the “cognitive network.”

Seed-to-voxel analyses of individual ROIs revealed both shared and dissociable patterns of functional connectivity, many of which recapitulate the anatomical networks observed in the DTI study, as well as the spatial architecture of canonical large-scale networks. The AI, together with the pACC, sACC, and the OFC, exhibited some similar connectivity aspects. These regions formed networks anchored in the AI and showed robust coupling with superior and middle frontal cortices as well as with anterior cingulate territories. However, despite these shared features, the overlap between some of these regions was relatively modest (e.g., AI–OFC), suggesting that their functional coupling should be interpreted as partial rather than strong. Accordingly, parietal contributions to these networks were more variable, involving medial parietal regions (as observed for the sACC) or lateral parietal areas (for the AI, pACC, and OFC). The vmPFC functional network resembled those of the AI, sACC, pACC, and OFC, but was distinguished by the absence of anterior insular and parietal contributions. Overall, the cortical topography and connectivity organization of the functional networks of these regions closely resembled the Default Mode Network in the case of the sACC and vmPFC, and the Ventral Attention Network in the case of the pACC, AI, and OFC. On the one hand, this dissociation indicates that the so-called emotional network spans multiple canonical systems, challenging a strictly unitary interpretation and instead suggesting functional heterogeneity within it. On the other hand, since both Default Mode and Ventral Attention networks are crucial for detecting behaviorally relevant external and internal stimuli, they may collectively support the areas of the emotional networks in the generation or inhibition of behavior, as well as the maintenance or suppression of vigilance and arousal (Menon 2011). In contrast, RO and FEF/vPMC showed preferential functional coupling with sensory and motor cortical territories and broader connectivity profiles, characterized by a common overlap of their functional networks with the Dorsal Attention Network. RO demonstrated functional coupling with precentral and postcentral cortices, characteristic of the canonical Sensorimotor Network, whereas the FEF/vPMC network encompassed superior frontal and parietal regions, closely resembling the canonical Frontoparietal and Ventral Attention Networks. Notably, the degree of similarity between RO and FEF/vPMC was relatively low compared to cross-cluster similarities (e.g., between FEF/vPMC and MFG or RO with pACC and AI), further suggesting that the premotor network may encompass functionally heterogeneous components rather than a tightly coherent network. This interpretation is consistent with: (1) the inclusion within the FEF/vPMC region of territories involved in both face and eye motor control, as well as attentional stimulus processing; and (2) the similarity between the connectivity pattern of RO and those of regions within the emotional cluster, such as the pACC and AI, in which intracortical microstimulation data elicited motor and visceromotor behaviors (Del Vecchio et al. 2024). Considering together these connectivity profiles support: for the RO a crucial role in voluntary motor control and for FEF/vPMC in the selection of sensory information and the control of movement-related responses, such as those involving the eyes, neck, and gaze (Corbetta and Shulman 2002; Vossel et al. 2014). Finally, the functional networks associated with the IFG and MFG displayed connectivity architectures resembling those of the Frontoparietal Network, particularly with regard to frontal territories, suggesting that these regions play a crucial role in integrating sensory stimuli with cognitive information to control appropriate motor outputs.

Together, the resting-state functional findings suggest that the areas constituting the various anatomically defined networks exhibit a more graded and overlapping organization across large-scale functional networks, rather than a strictly cluster-based partition.

### The emotional network

According to tractography results (Fig. 10), we designated the emotional network as the set of areas comprising the sACC, pACC, vmPFC, OFC, and AI, as these regions are not only extensively interconnected with one another but also contribute to the control and regulation of emotions, socio-emotional responses, and interoception (Ongur & Price, 2000; Vogt 2005; Drevets et al. 2008; Haber et al. 2009; Laxton et al. 2013). The areas of this network are connected through three main bundles: CB, AC, and the UF. Specifically, the CB links the pACC, the sACC, and the vmPFC to one another. This bundle is a major white matter tract connecting frontal, parietal, temporal, and limbic regions (Bubb et al. 2018) and is known to play a key role in integrating emotional, memory, and attentional processes (Catani & Thiebaut de Schotten, 2008). Stimulation of the CB has been shown to produce spontaneous laughter, enhance positive emotional experiences, reduce anxiety, and modulate pain perception (Bijanki et al. 2019). The CB can also convey emotion-related signals to the pACC and sACC from the vmPFC, which in turn can receive multisensory inputs with emotional valence from the OFC, known to be involved in the evaluation of sensory stimuli, particularly their reward or punishment value (Rolls 2023).

The vmPFC is connected to the OFC also via the AC, which also connects the OFC with the sACC. The AC is the most ancient interhemispheric commissure and connects one to another the amygdala, temporal, frontal, and occipital lobes (Fenlon et al. 2021). It is crucial for bilateral integration of emotional and sensory information (Fenlon et al. 2021). In fact, lesion studies in both animals and humans indicate that damage of the AC impairs memory, vision and olfaction, as well as emotional processing (Sisodiya et al., 2001; Mitchell et al. 2002; Fenlon et al. 2021). On the basis of the current knowledge on the AC, we speculated that, as far as emotional processing is concerned, the AC may support the consolidation of negative emotional stimuli, linking the sACC with the vmPFC and OFC, and also contributing to reward and punishment integration via downstream effects on amygdala regulation.

The UF connects the AI and the OFC one to another and with the sACC and the vmPFC; this bundle is a hook-shaped white matter tract known to connect the anterior temporal lobe (including the amygdala and temporal pole) with the OFC and vmPFC (Petrides and Pandya 2007; Schmahmann et al., 2007; Thiebaut de Schotten et al., 2012). Although its precise function is not fully understood, it is known to be involved in emotion, memory, language, and social behavior (Gaffan and Wilson 2008; Ross 2008), and is thought to support the temporo-amygdala-orbitofrontal network, which is crucial for processing socially relevant stimuli such as facial expressions (Catani et al., 2012). It has been recently suggested that UF plays a broad integrative role in enabling mnemonic associations - including those involving faces - to influence behaviour via interactions with the lateral orbitofrontal cortex, which contributes to valence-based modulation of decision-making (Von Der Heide et al. 2013). In line with this hypothesis, we suggest that both UF and AC may transmit information endowed with emotional content, such as emotional faces, from the AI and OFC, respectively, to the vmPFC. Within the vmPFC, these visual inputs - also carrying social-value information - can be integrated with information coming from the entorhinal cortex and hippocampus, functionally connected to the vmPFC, to form episodic memory traces of the observed faces (Hiser and Koenigs 2018; Rolls 2023). Through the CB, the vmPFC can relay this integrated information to the ACC, where it can be utilized to generate adaptive behavioral responses, such as the emotional motor contagion. In particular, to produce positive behavior in the case of pACC, whose stimulation in fact produces spontaneous laughter (Caruana et al., 2020; Bijanki et al. 2019), and negative one, in the case of sACC, known to be activated during the automatic and implicit processing of unpleasant stimuli and involved in pathological depression (Laxton et al. 2013).

The rsfMRI findings related to the territories of the emotional network are consistent with DTI results. Indeed, their functional networks show specific overlap in the anterior insula and cingulate cortices, consistent with the idea that they serve as hub regions facilitating communication among the territories comprising this network. As described above, compared with canonical resting-state networks (Yeo et al. 2011), the regions of the emotional network exhibit connectivity patterns closely resembling those of the Ventral Attention Network (VAN) for the pACC, AI, and OFC and those of the Default Mode Network (DMN) for the sACC and vmPFC. Different studies describe the role of VAN in the detection of behaviorally relevant stimuli, particularly when they are unexpected (Corbetta and Schulman, 2002), in mediating the interaction between internal representations and external sensory events (Petersen and Posner 2012), and in switching between external sensory information and internal memory contents, allowing for flexible behavior (Nobre and Gresch, 2025. The DMN is well known to play a crucial role in emotional perception, by integrating internal states, autobiographical memory, and social cognition with pleasant and unpleasant experiences (Buckner et al. 2008; Sambuco 2024).

In agreement with both their anatomical and functional connections, electrical stimulation of all the regions of the emotional network, but OFC, typically triggers positive or negative emotional responses, or autonomic responses (Caruana et al. 2018; Caruana et al., 2020; Pelliccia et al. 2023; Del Vecchio et al. 2024; Yih et al. 2019; Pantis et al. 2025; Tian et al. 2025).

In summary, altogether our data suggest that when a behaviorally relevant and potentially unexpected stimulus, such as an emotional face, is observed, the emotional network may reflect a remodulation of the observer’s internal state by integrating it with incoming external sensory cues, ultimately leading to an appropriate behavioral response, which may involve the generation, or inhibition, of emotional contagion.

### The premotor network

According to tractography results (Fig. 10), the premotor cluster is constituted by the rolandic operculum (RO) and the FEF/ventral premotor cortex (FEF/vPMC) that are connected by the aslant tract (FAT). This result was obtained exclusively using the probabilistic approach. The discrepancy likely arises from the fact that, with the deterministic approach, this portion of white matter corresponds to a region of fiber crossing that is difficult to resolve with deterministic algorithms. However, we are confident in the existence of this tract based on the possibility of comparing these connections with those of homologous regions in the monkey, where connectivity has been investigated using neuronal tracers, the gold standard technique for determining whether two regions are connected. In fact, the monkey homolog of the rolandic operculum is known to be strongly connected with the ventral premotor cortex (vPMC), likely via a white matter pathway homologous to the frontal aslant tract (Matelli et al. 1986; Cipolloni and Pandya 1999; Gerbella et al. 2011, 2016). The FAT is a system of fibers that links the superior frontal gyrus with the pars opercularis of the inferior frontal gyrus (IFGop) (Tagliaferri et al. 2023, 2024), some projections of which also reach the pars triangularis of the IFG and the inferior region of the precentral gyrus (Catani et al., 2012), including RO. The FAT has been found to connect key regions involved in the production of non-emotional laughter as the pre-SMA and RO (Gerbella et al. 2021). Damage to this tract can result in Foix-Chavany-Marie syndrome, an impairment of voluntary control of certain facial and pharyngeal movements (e.g., laughing, coughing; Martino et al. 2012; Chernoff et al. 2019), and its stimulation can provoke alteration of speech, a motor behavior sharing many motor structures with volitional laughter (Corrivetti et al. 2019; Belyk and McGettigan 2022). In addition, a recent study found that an increase in the volume of this tract is positively correlated with the severity of motor signs in stuttering, supporting its role in functions such as inhibitory control and conflict monitoring (Neef et al. 2022).

Resting-state fMRI results indicate that the RO functional network, encompassing precentral and postcentral cortices, largely corresponds to the canonical Sensorimotor Network, a large-scale system primarily involved in the planning, execution, and monitoring of voluntary movements, as well as in processing sensory information from the body (Yeo et al., 2011). Accordingly, high-frequency electrical stimulation (HF-ES) on this region elicited sensorimotor responses in the face and mouth (Del Vecchio et al. 2024; Caruana et al. 2016; 2020; Mălîia et al. 2018). The RO functional network showed also some overlap with the Dorsal Attention Network (DAN), that is known to be involved in top-down, goal-directed selection of attended stimuli and responses (Petersen and Posner 2012). In line with the role of the DAN in mediating the voluntary orienting of spatial attention and sustaining attention to task-relevant information (Corbetta and Shulman 2002). Similarly, also the FEF/vPMC network exhibited partial overlap with the DAN, consistent with its role in voluntary orienting of spatial attention and sustaining attention to task-relevant information (Corbetta and Shulman 2002). Both RO and FEF/vPMC networks also overlapped with the VAN, suggesting that these regions are modulated not only by top-down control but also by bottom-up responses to unattended stimuli. Unlike the RO, the functional coupling of the FEF/vPMC involved the Sensorimotor Network only marginally and primarily engaged parieto-frontal regions that largely overlap with territories of the Frontoparietal Network, which is known to mediate the goal-directed selection of sensory information through flexible interactions with both the DAN and VAN (D'Esposito and Postle 2015), thereby optimizing perceptual processing in accordance with current behavioral goals (Corbetta and Shulman 2002; Vossel et al. 2014). Accordingly, HF-ES of the FEF/vPMC elicited ocular and contralateral neck movements, as well as eyelid clonicity, suggesting that its role in controlling gaze and neck movements is specifically related to attention-demanding stimuli, such as facial expressions (Del Vecchio et al. 2024). Interestingly, both areas of the premotor network are not only involved in controlling voluntary facial and eye movements but also during action observation (Grosbras et al. 2005; Caspers et al. 2010). In particular, the vPMC is a crucial node of the mirror neuron system (Diehl et al. 2022; Tsao et al. 2008; Fogassi and Simone 2013), in line with its well-known recruitment during passive observation of emotional expressions (Hamzei et al. 2016; Hsu et al. 2022; Krautheim et al., 2020; Li et al. 2023; Rymarczyk et al. 2018). Also, the RO is activated during both the observation and execution of positive and negative expressions (Carr et al. 2003; Leslie et al. 2004). Accordingly, both functional networks of FEF/vPMC and RO showed activated voxels in key areas - including other premotor cortices, M1, and parietal regions - within the parieto-frontal mirror circuits (Rizzolatti et al. 2014).

Summarizing, altogether our data are compatible with the hypothesis that the FAT may enable the RO, via attentional information and stimulus detection from FEF/vPMC, to produce voluntary facial expressions and eye movements based on attentional cues and independently of a real affective experience, in accord to the notion that unlike spontaneous facial expressions the voluntary ones serve strategic communicative functions, guiding social interactions and signaling intent (Provine 2016; Wood and Niedenthal 2018). In addition, the connections of the FEF/vPMC with the IFG, well known to be involved in language, suggests that this region may contribute to the conversational aspects of voluntary facial expressions and eye movements, which often occur automatically during social interactions to better convey the speaker’s communicative intent (Caruana and Palagi 2025).

Finally, it is noteworthy that, although our data suggest that the RO and FEF/vPMC appear primarily involved in the production and observation of voluntary facial expressions, it cannot be excluded that they may also reflect the association between facial imitation accuracy and interpersonal emotional regulation. In other words, these regions may contribute to interpersonal emotional regulation through the production of pure motor behaviors, such as facial imitation and eye movements modulation, able to indirectly influence the affective state of the subject (Wang et al., 2024).

### The cognitive network

Possible resources of visual inputs to the premotor network are the two lateral prefrontal areas IFG and MFG, among which the IFG can be considered a nexus connecting the premotor cluster with the emotional-limbic one via the IFOF (Fig. 10). The IFOF is a long-range white matter tract traverses the external capsule to link occipital, parietal, and temporal cortices with lateral and ventral frontal regions, enabling multimodal integration across visual, semantic, and affective networks (De Benedectis, 2021). Electrical stimulation at its orbitofrontal terminations can elicit complex visual hallucinations (Andelman-Gur, 2020). Clinically, IFOF lesions or disconnections impair emotional-face recognition and are implicated in Capgras syndrome, congenital prosopagnosia, and autism severity (Philippi et al. 2009; Bobes et al., 2016; Kilroy et al. 2022). In accordance with its strategic anatomical position, the IFOF is considered able to mediate interactions between the ventral visual stream (responsible for objects and face recognition) and cortical regions related to emotions (Philippi et al. 2009), such as the areas of the emotional network. Specifically, our data suggest that the IFOF, through its occipital connections, may carry non-emotional visual information to IFG, OFC, and AI, providing these regions with pictorial/semantic information about observed facial expression. Along this pathway, we speculate that the IFG may serve as a hub where, on one hand, visual information is integrated to interpret observed expressions within complex social contexts, guiding appropriate communicative interactions, and potentially inhibiting contextually inappropriate behaviors, and, on the other hand, is relayed to the AI and OFC. Accordingly, resting‐state fMRI revealed that IFG coactivates within the Frontoparietal network, that, as described above, underpin goal‐directed behavior and flexible cognitive control, in line with the possible role of this region in integrating multisensory contextual cues with their emotional counterparts. In agreement with this hypothesis, the IFG has been shown to be active during both passive and non-passive observation of both voluntary and spontaneous emotional expressions (Del Vecchio et al. 2024; Dricu and Frühholz 2016; Fusar-Poli et al., 2009; Sabatinelli et al. 2011; Jabbi & Keysers, 2008). In addition, lesion studies indicate that damage to this frontal region affects the recognition of facial expressions (Uono et al. 2017; Adolphs et al. 2000; Dal Monte et al. 2013; Philippi et al. 2009) and of prosody (Mattavelli et al., 2019).

As mentioned above an additional resource of visual input to the premotor network is the MFG; similarly to IFG, also this region is activated during the recognition of various types of emotional faces, such as happy, angry, and fearful expressions (Haller et al., 2018); however, intracranial stimulation of this region did not elicit overt motor or visceral responses, suggesting a modulatory rather than a primary motor role in emotional processing (Del Vecchio et al. 2024), in line with studies proposing a role for the MFG in top-down attentional control and incongruity detection during facial-expression processing (Japee et al. 2015; Chan et al. 2012). The MFG is connected to areas of the premotor network via the SLF, a fiber bundle linking frontal, parietal, and temporal perisylvian regions, thereby enabling integration between attentional and motor–premotor networks (Catani et al., 2012). Lesions of the SLF impair executive control and the recognition of facial expressions, particularly happiness (Ioannucci et al. 2020; Van den Berg et al. 2021), and tractography studies have linked SLF integrity to accurate decoding of emotional faces (Tükel et al. 2017). Therefore, it is tempting to speculate that the SLF may provide the areas of the premotor network with higher-order multisensory information about relevant emotional stimuli (facial expressions), supporting also in this case the monitoring and modulation of appropriate behavioral responses. Accordingly, the MFG coactivates within the Frontoparietal network and, to a lesser extent, the DAN, further highlighting its potential role in the top-down modulation of attention and gaze toward salient stimuli, such as emotional facial expressions, based on the contextual cues in which we find ourselves (Del Vecchio et al. 2024).

### Limitations

Some limitations of the present study should be acknowledged. First, the connectivity analyses were restricted to a set of a priori selected ROIs derived from our previous intracranial electrophysiological study of the emotional face processing. While this approach allowed us to focus specifically on regions that showed direct electrophysiological sensitivity to emotional facial expressions, it necessarily limits the scope of the analysis and does not exclude the possible involvement of additional regions not included in the present ROI set. Second, the original identification of responsive sites was based on intracranial recordings, which are inherently affected by sampling constraints determined by clinical implantation strategies. Therefore, the regions examined here cannot be considered an exhaustive representation of the broader network involved in facial emotion processing, despite the dense sampling of our study, which substantially reduces this issue. Fourth, the three types of analyses were carried out on different sets of subjects, and although this approach reflects the availability of suitable data for each modality rather than a specific methodological preference, it did not allow for a direct comparison of the studied territories within a single subject. Finally, although the combination of tractography and resting-state functional connectivity provides complementary information about large-scale brain organization, both approaches remain correlational in nature and do not allow direct inferences about the directionality or causal role of the identified connections. For this reason, the functional interpretations presented here therefore highlight plausible roles for these networks, which can be further explored in future task-based or behavioral studies.

## Conclusion

The present study outlines a three-component synergistic organization of frontal and insular regions involved in the perception of emotional facial expressions, comprising emotional, motor, and cognitive networks. Structural connectivity analyses indicate that these networks are supported by distinct pathways, with regions such as the OFC and IFG exhibiting hub-like connectivity across networks. These findings highlight a distributed architecture in which different subnetworks are structurally and functionally interlinked, supporting parallel contributions to facial emotion processing. Importantly, while the anatomical organization suggests a relatively degree of segregation into distinct components, functional connectivity patterns point to a more graded and partially overlapping organization across large-scale functional networks, rather than a strict cluster-based partition. While the precise functional roles of these networks remain to be further clarified, the identified connectivity patterns offer a biologically plausible framework for understanding how emotional, motor, cognitive, and attentional processes may interact during emotion perception. These results provide a foundation for future studies to explore causal and task-dependent dynamics. Overall, our data support a multi-component rather than unitary system for facial emotion recognition, emphasizing the complementary contributions of distinct frontal and insular circuits.

## Supplementary Information

Below is the link to the electronic supplementary material.

Supplementary Material 1

## Data Availability

Data will be made available on request.
