# Supplementary material for: Inside the frontal face network: multimodal evidence for distinct emotional and motor resonance circuits
Source: Brain Struct Funct. 2026 May 28;231(5):72. doi: 10.1007/s00429-026-03125-5 (PMC13219183; doi:10.1007/s00429-026-03125-5)
Supplement: Supplementary file 1 — Supplementary Material 1 [file 429_2026_3125_MOESM1_ESM.docx]

**Supplementary Material**

**Deterministic analysis**

*Fear condition right hemisphere*

In the right hemisphere, the frontal regions encoding fearful expressions are connected by the inferior frontal occipital fasciculus (IFOF), the anterior commissure bundle, the cingulum fasciculus, and in the left hemisphere. Specifically, the IFOF connects the second seed in the anterior insula with the fourth seed of the inferior frontal gyrus (IFG-4) and the anterior commissure connects the second seed of the subgenual cingulate cortex (sACC-2) with the third seed in the ventromedial prefrontal cortex (vmPFC-3). The cingulum fasciculus connects the third seed in the pregenual anterior cingulate cortex (pACC-3) with the second seed in the subgenual cingulate cortex (sACC-2). In addition to these, the superior longitudinal fasciculus (SLF) connects the second seed in the middle frontal gyrus (MFG-2) with the first seed in inferior frontal junction (vPMC-1), and the first seed in the middle frontal gyrus (MFG-1) with the first seed in the Rolandic operculum (RO-1), respectively (Figure 4A). The same bundle also connects the first seed of the inferior frontal junction (IFJ-1) with the fourth seed of the inferior frontal gyrus (IFG-4) (Supplementary Table 1).

**Supplementary Table 1**

Tracts connecting ROIs based on the frontal regions activated by fearful expressions in the right hemisphere.

| **FEAR LEFT** | | | | | | | | | | | |
| --- | --- | --- | --- | --- | --- | --- | --- | --- | --- | --- | --- |
| **ROIs** | **OFC2** | **vmPFC3** | **MFG1** | **MFG2** | **AI2** | **AI3** | **sACC2** | **pACC3** | **vPMC1** | **IFG4** | **RO1** |
| **OFC2** |  |  |  |  |  |  | AC |  |  |  |  |
| **vmPFC3** |  |  |  |  |  |  | AC |  |  |  |  |
| **MFG1** |  |  |  |  |  |  |  |  |  |  | SLF |
| **MFG2** |  |  |  |  |  |  |  |  | SLF |  |  |
| **AI2** |  |  |  |  |  |  |  |  |  | IFOF |  |
| **sACC2** | AC | AC |  |  |  |  |  | CB |  |  |  |
| **pACC3** |  |  |  |  |  |  | CB |  |  |  |  |
| **vPMC1** |  |  |  | SLF |  |  |  |  |  | AF/SLF |  |
| **IFG4** |  |  |  |  | IFOF |  |  |  | AF/SLF |  |  |
| **RO1** |  |  | SLF |  |  |  |  |  |  |  |  |

*Fear condition left hemisphere*

In the fear condition, we found that in the left hemisphere that the IFOF connects the anterior insula (AI-3) with two seeds in the inferior frontal gyrus (IFG-1 and IFG-2) and one in the orbitofrontal cortex (OFC-3) (Figure 3A). The IFOF also connects the first and second seeds of the orbitofrontal cortex (OFC-1 and OFC-3). The anterior commissure bundle connects the first seed of the subgenual anterior cingulate cortex (sACC-1) with the second seed in the orbitofrontal cortex (OFC-2). In addition, the cingulum fasciculus connects the vmPFC-1 with the sACC-1 that in turn is connected to the sACC-2 trough the anterior commissure (Supplementary Table 2).

**Supplementary Table 2**

Tracts connecting ROIs based on the frontal regions activated by fearful expressions in the left hemisphere.

| **FEAR LEFT** | | | | | | | | | | |
| --- | --- | --- | --- | --- | --- | --- | --- | --- | --- | --- |
| **ROIs** | **OFC1** | **OFC2** | **OFC3** | **vmPFC1** | **AI3** | **sACC1** | **sACC2** | **vPMC2** | **IFG1** | **IFG2** |
| **OFC1** |  | UF | UF |  |  |  |  |  |  |  |
| **OFC2** |  |  |  |  |  | AC | AC |  |  |  |
| **OFC3** | IFOF |  |  |  | IFOF |  |  |  |  |  |
| **vmPFC1** |  |  |  |  |  | CB |  |  |  |  |
| **AI3** |  |  | IFOF |  |  |  |  |  | IFOF | IFOF |
| **sACC1** |  | AC |  | CB |  |  | AC |  |  |  |
| **sACC2** |  |  |  |  |  | AC |  |  |  |  |
| **vPMC2** |  |  |  |  |  |  |  |  |  |  |
| **IFG1** |  |  |  |  | IFOF |  |  |  |  |  |
| **IFG2** |  |  |  |  | IFOF |  |  |  |  |  |

* tracts observed only using the probabilistic approach.

*Smile condition right hemisphere*

The seeds in the right hemisphere showed that the cingulum fasciculus connects the first three seeds in the pregenual anterior cingulate cortex (pACC-1, pACC-2 and pACC-3) with the second seed in the ventromedial prefrontal cortex (vmPFC-2) (Supplementary Table 3). In the left hemisphere, instead, the cingulum fasciculus connects the first and the second seeds of the pregenual anterior cingulate (pACC-1 and pACC-2) with the first seed of the subgenual anterior cingulate cortex (sACC-1) (Supplementary Table 4). Furthermore, this latter seed in the right subgenual anterior cingulate cortex (sACC-1) is connected through the anterior commissure to the second seed of the ventromedial prefrontal cortex (vmPFC-2) of the left hemisphere (Supplementary Table 3).

**Supplementary Table 3**

Tracts connecting ROIs based on the frontal regions activated by smile expressions in the right hemisphere.

| **SMILE RIGHT** | | | | | | | | | | | |
| --- | --- | --- | --- | --- | --- | --- | --- | --- | --- | --- | --- |
| **ROIs** | **OFC 1** | **OFC2** | **vmPFC2** | **AI1** | **AI3** | **AI4** | **vPMC1** | **pACC1** | **pACC2** | **pACC3** | **IFG2** |
| **OFC 1** |  |  | UF |  |  |  |  |  |  |  |  |
| **OFC2** |  |  | UF |  |  |  |  |  |  |  |  |
| **vmPFC2** | UF | UF |  |  |  |  |  | CB | CB | CB |  |
| **AI4** |  |  |  |  |  |  |  |  |  |  | IFOF |
| **vPMC1** |  |  |  |  |  |  |  |  |  |  |  |
| **pACC1** |  |  | CB |  |  |  |  |  |  |  |  |
| **pACC2** |  |  | CB |  |  |  |  |  |  |  |  |
| **pACC3** |  |  | CB |  |  |  |  |  |  |  |  |
| **IFG2** |  |  |  |  |  | IFOF |  |  |  |  |  |

*Smile condition left hemisphere*

In the left hemisphere, the arcuate fasciculus (AF) connects the fifth seed of the anterior insula (AI-5) with the fourth and second seeds in the inferior frontal gyrus (IFG-4 and IFG-2) (Supplementary Table 4). In the right hemisphere, instead, the IFOF connects the fourth seed in the anterior insula (AI-4) with the second seed of the inferior frontal gyrus (IFG-2) (Figure 5C). Finally, always in the same hemisphere, the uncinate fasciculus (UF) connects the first and the second seeds in the orbitofrontal cortex (OFC-1 and OFC-2) with both the first seed in the anterior insula (AI-1) and the second seed in the ventromedial prefrontal cortex (vmPFC-2) (Supplementary Table 4).

**Supplementary Table 4**

Tracts connecting ROIs based on the frontal regions activated by smile expressions in the left hemisphere.

| **SMILE LEFT** | | | | | | | |
| --- | --- | --- | --- | --- | --- | --- | --- |
| **ROIs** | **AI5** | **sACC1** | **pACC1** | **pACC2** | **vPMC1** | **IFG2** | **IFG4** |
| **AI5** |  |  |  |  |  | AF | AF |
| **sACC1** |  |  | CB | CB |  |  |  |
| **pACC1** |  | CB |  |  |  |  |  |
| **pACC2** |  | CB |  |  |  |  |  |
| **IFG2** | AF |  |  |  |  |  |  |
| **IFG4** | AF |  |  |  |  |  |  |

###

### **Probabilistic analysis**

Ball-and-stick analysis confirmed that all the white matter tracts observed in the deterministic analysis can also be traced using the probabilistic approach. In particular, this analysis showed that, in almost all subjects from both datasets, the tracts described in the deterministic analysis can be identified. Although nearly all pairs of ROIs connected with DSI Studio are also connected using the probabilistic approach, there are very few exceptions, that are the following ROI pairs: vPMC1–RO3, vmPFC1–sACC1, vmPFC1–sACC2, and vmPFC2–pACC1. In addition, the probabilistic analysis, being less restrictive with respect to the deterministic one, showed further connections not observed with this latter analysis. Specifically, ball-and-stick analysis showed that: 1) IFOF connects the seeds of the OFC with the seeds of the anterior insula in both hemispheres of the largely majority of the subjects in both datasets (Supplementary Tables 5 and 6); 2) the frontal aslant tract (FAT) connects the FEF/vmPMC with RO in at least two pair of ROIs considered (FEF/vPMC2-RO2 and FEF/vPMC1-RO1) .

**Supplementary Table 5**

Tracts connecting ROIs on our dataset

| Hemisphere | Left | |  | Right | |
| --- | --- | --- | --- | --- | --- |
| Tract | **Subjects** | **Average of streamlines** | **Tract** | **Subjects** | **Average of streamlines** |
| AI3 -IFG1 | 8\12 | 0.63 | **AI4_IFG2** | 12\12 | 0.83 |
| AI3 -IFG2 | 12\12 | 0.83 | **IFG4_AI2** | 12\12 | 0.66 |
| AI5 -IFG2 | 12\12 | 0.75 | **IFG4_vPMC1** | 12\12 | 0.58 |
| AI5 -IFG4 | 12\12 | 0.75 | **MFG1_RO1** | 11\12 | 0.5 |
| OFC2-AI3 | 9\12 | 0.6 | **MFG2-RO1** | 10\12 | 0.5 |
| OFC2-sACC1 | 0\12 | / | **MFG2-vPMC1** | 11\12 | 0.58 |
| OFC1-AI3 | 10\12 | 0.5 | **OFC1_AI1** | 12\12 | 1.00 |
| OFC2-sACC2 | 11\12 | 0.75 | **OFC1_AI2** | 9\12 | 0.50 |
| OFC3 -AI3 | 12\12 | 0.5 | **OFC1_AI3** | 9\12 | 0.41 |
| FEF/vPMC1-RO3 | 0\12 | / | **OFC1_AI4** | 11\12 | 0.66 |
| FEF/vPMC2-RO2 | 9\12 | 0.6 | **OFC1-vmPFC2** | 12\12 | 0.41 |
| sACC1-pACC1 | 12\12 | 0.5 | **OFC2_vmPFC2** | 12\12 | 0.50 |
| sACC1-pACC2 | 11\12 | 0.6 | **OFC2-AI1** | 12\12 | 0.83 |
| vmPFC1 -sACC1 | 12\12 | 0.25 | **OFC2-AI2** | 11\12 | 0.58 |
| vmPFC1-sACC1 | 0\12 | / | **OFC2_AI3** | 10\12 | 0.58 |
|  |  |  | **OFC2-AI4** | 12\12 | 0.83 |
|  |  |  | **vmPFC2-pACC2** | 10\12 | 0.58 |
|  |  |  | **vmPFC2-pACC3** | 9\12 | 0.58 |
|  |  |  | **vmPFC3-sACC2** | 12\12 | 0.50 |
|  |  |  | **FEF/vPMC1-RO1** | 12\12 | 0.58 |
|  |  |  | **sACC2-pACC3** | 12\12 | 0.50 |
|  |  |  | **vmPFC1-MFG2** | 0\12 | / |
|  |  |  | **vmPFC2-pACC1** | 0\12 | / |

**Supplementary Table 6**

Tracts connecting ROIs on WU–Minn dataset

| Hemisphere | Left | |  | Right | |
| --- | --- | --- | --- | --- | --- |
| Tract | **Subjects** | **Average of streamlines** | **Tract** | **Subjects** | **Average of streamlines** |
| AI3 -IFG1 | 1\4 | 0.3 | **AI4-IFG2** | 4\4 | 1.00 |
| AI3 -IFG2 | 4\4 | 1.00 | **IFG4-AI2** | 4\4 | 1.00 |
| AI5 -IFG2 | 1\4 | 0.25 | **IFG4-vPMC1** | 4\4 | 1.00 |
| AI5 -IFG4 | 3\4 | 0.75 | **MFG1-RO1** | 2\4 | 0.5 |
| OFC2-AI3 | 4\4 | 0.75 | **MFG2-RO1** | 2\4 | 0.50 |
| OFC2-sACC1 | 0\4 | / | **MFG2-vPMC1** | 4\4 | 0.75 |
| OFC1-AI3 | 4\4 | 1.00 | **OFC1-AI1** | 4\4 | 1.00 |
| OFC2-sACC2 | 4\4 | 1.00 | **OFC1-AI2** | 3\4 | 1.00 |
| OFC3 -AI3 | 3\4 | 0.5 | **OFC1-AI3** | 0\4 |  |
| FEF/vPMC1-RO3 | 0\4 | / | **OFC1-AI4** | 4\4 | 1.00 |
| FEF/vPMC2-RO2 | 4\4 | 1.00 | **OFC1-vmPFC2** | 4\4 | 0.75 |
| sACC1-pACC1 | 3\4 | 0.75 | **OFC2_vmPFC2** | 4\4 | 1.00 |
| sACC1-pACC2 | 1\4 | 0.25 | **OFC2-AI1** | 4\4 | 1.00 |
| vmPFC1 -sACC1 | 2\4 | 0.5 | **OFC2-AI2** | 4\4 | 1.00 |
| vmPFC1-sACC1 | 0\4 | / | **OFC2-AI3** | 1\4 | 0.25 |
|  |  |  | **OFC2-AI4** | 3\4 | 0.75 |
|  |  |  | **vmPFC2-pACC2** | 2\4 | 0.5 |
|  |  |  | **vmPFC2-pACC3** | 3\3 | 0.75 |
|  |  |  | **vmPFC3-sACC2** | 0\12 |  |
|  |  |  | **vPMC1-RO1** | 4\4 | 0.75 |
|  |  |  | **sACC2-pACC3** | 1\4 | 0.25 |
|  |  |  | **vmPFC1-MFG2** | 0\4 | / |
|  |  |  | **vmPFC2-pACC1** | 0\4 | / |

#
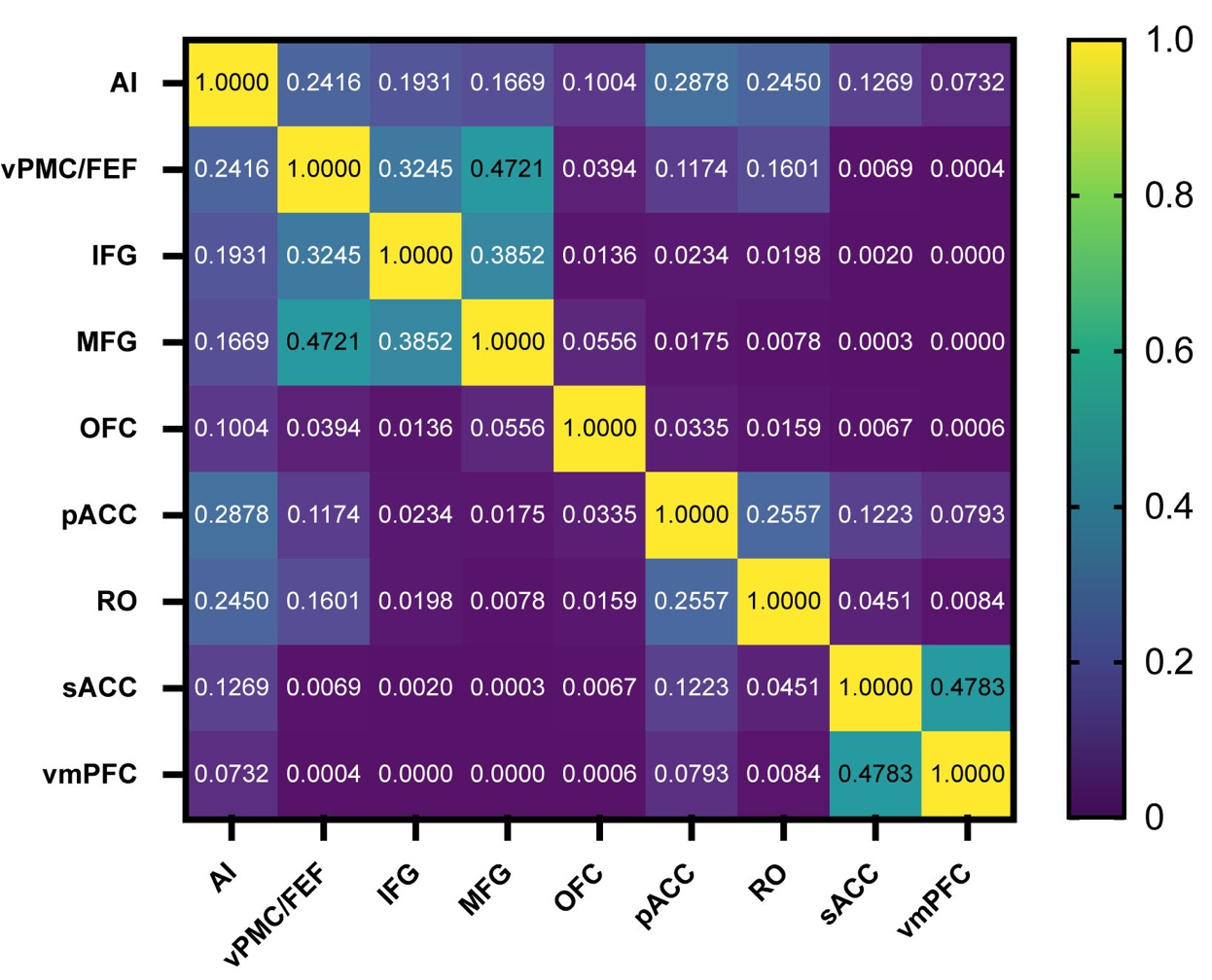


***Supplementary Figure 1.*** *Dice coefficients showing how much the functional network here identified overlapped one to another.*
